# Food craving in a cultural context: behavioral and electrophysiological evidence from Indian and Western food sets

**DOI:** 10.3389/fpsyg.2026.1940439

**Published:** 2026-10-28

**Authors:** Subhajit Das, Avishek Chatterjee, Satyaki Mazumder, Koel Das

**Affiliations:** 1 Department of Mathematics and Statistics, Indian Institute of Science Education and Research Kolkata, Mohanpur, India; 2 GlaxoSmithKline plc, Bangalore, India

**Keywords:** cultural influence, food craving, Indian population, indigenous and Western food, multisensory exposure, neural correlates

## Abstract

**Introduction:**

Food craving is a ubiquitous experience, often shaped by cultural familiarity. Although craving may be considered a culture-bound construct, its neural and behavioral underpinnings across food cultures remain limited. Here, we examined the effects of multisensory food exposure on craving in an Indian population, comparing Indian and Western foods across sweet and savory categories.

**Methods:**

In Experiment 1 (Indian Food Craving; IFC), participants viewed Indian sweet and savory food images before and after multisensory exposure, reporting desire and willingness-to-pay (bid) ratings along with Food Craving Questionnaire-State (FCQ-S) scores. EEG was recorded using a 64-channel system. In Experiment 2 (Western Food Craving; WFC), participants completed a similar paradigm using Western food items.

**Results:**

Multisensory exposure significantly increased FCQ-S scores in both experiments, indicating successful craving induction. Behaviorally, desire and bid ratings increased for both sweet and savory Indian foods, whereas for Western foods, increases were observed only for sweet items. ERP analyses revealed increased P200 and N200 amplitudes following exposure to Indian sweet foods, while LPP amplitudes increased for savory foods across both Indian and Western food categories. Complementary MVPA revealed enhanced pre- vs. post-exposure classification for sweet foods during the early (0–300 ms) window for both Indian and Western foods, whereas both sweet and savory foods showed significant classification during the later(300–600 ms) window.

**Discussion:**

Together, these findings suggest that multisensory exposure modulates food-cue processing in a stimulus- and time-dependent manner, with possible contributions from attentional processes. Overall, the findings highlight the influence of food type and cultural context on craving-related neural and behavioral responses.

## 1 Introduction

Food craving, defined as a strong desire for specific foods, is a common experience typically directed toward highly palatable, energy-dense items rich in fat and sugar (Lafay et al., 2001). Craving is not solely driven by hunger but is closely linked to emotional and motivational states (Hill, 2007; Reents et al., 2020). Experimental studies show that exposure to food-related cues (e.g., images, smells) reliably increases subjective desire and influences food valuation and consumption behavior (Boswell and Kober, 2016; Ferriday and Brunstrom, 2011).

Neurocognitive research demonstrates that food cues engage reward-related brain regions, including the orbitofrontal cortex, striatum, insula, and amygdala (Pelchat, 2002; Hare et al., 2009). Electrophysiological studies further reveal the temporal dynamics of these processes: early ERP components (e.g., P200, N200) reflect rapid attentional orienting, whereas later components (e.g., P300, LPP) index motivational salience and sustained attention (Blechert et al., 2014; Carbine et al., 2018). Together, these findings suggest that food craving may involve multiple stages of processing, ranging from relatively early attentional responses to later motivational and evaluative mechanisms.

An important but comparatively less studied influence on food craving is culture. Eating behavior is embedded in cultural contexts that shape food preferences, culinary practices, and the emotional meanings attached to specific foods. Consequently, responses to food cues may depend not only on their sensory or nutritional properties but also on an individual's familiarity and experience with culturally specific foods. Systematic study of cultural effect in food craving has received little attention in cognitive neuroscience (Rozin, 1996a, 2013). Much of the existing literature is based on Western populations and stimuli, raising concerns about generalizability and ecological validity. Eating behavior is deeply embedded in cultural contexts that shape food preferences, meanings, and habitual practices (Rozin, 1996b, 2013). Studies comparing cross-cultural effect of food craving between US and Spanish population found gender effect in food choices (sweet/savory) and cultural effect in chocolate craving (Zellner et al., 1999; Osman and Sobal, 2006). In another questionnaire study, contrary to Western population, an Egyptian population found intensified craving for savories rather than sweet items (Parker et al., 2003). Similarly, cross-cultural research has shown that dietary customs and food availability shape taste preferences across populations, indicating that sensory preferences adapt to culturally specific food environments (Sorokowska et al., 2017). Recent cross-cultural studies emphasized the possible role of culture in modulating the neural response to food cues (Nymana and Usun, 2025) while food familiarity was shown to be a guiding factor for sensory liking (Torrico et al., 2019).

Indigenous or culturally familiar foods play a central role in this context, as they are closely tied to cultural identity and often carry strong emotional significance. However, globalization has increased exposure to Western dietary patterns in many non-Western societies, contributing to a “nutrition transition” toward processed foods high in sugar, fat, and refined carbohydrates (Popkin, 2017; Pingali, 2007). India provides a particularly relevant setting to study this interaction. Rapid urbanization and economic growth have altered dietary patterns, with rising consumption of ultra-processed and Western-style foods, especially among urban populations (Tak et al., 2022; Law et al., 2019). At the same time, traditional dietary practices remain culturally meaningful and continue to shape food preferences and eating behavior (Samaddar et al., 2020; Das and Priya, 2022). These findings suggest that many individuals in India navigate a hybrid food environment, where culturally familiar foods coexist with increasingly accessible Western foods.

Food perception relies on the integration of visual, olfactory, gustatory, and cognitive information, and exposure to food-related sensory cues can substantially alter craving and attentional processing. Availability and habitual exposure are important determinants of both behavioral and neural responses to food cues. Repeated consumption strengthens learned associations between food cues and reward, thereby increasing their motivational salience, although prolonged exposure may also produce habituation under certain conditions. In addition, food availability influences reward expectancy, with anticipated availability enhancing activation in reward-related brain regions and making food cues more motivationally significant when consumption is expected (Blechert et al., 2016). Culturally familiar and frequently consumed foods generally evoke stronger behavioral responses and greater engagement of reward and attentional networks than unfamiliar foods, reflecting the influence of long-term dietary experience on food cue processing. Thus, culturally familiar foods may differ from less familiar foods not only in their symbolic or cultural significance but also in the strength of previously acquired sensory-reward associations. Recent evidence suggests that multisensory exposure to sweet or savory food cues not only increases the desire for the exposed food category but can also generalize to foods sharing similar sensory characteristics (Cho and Hong, 2025). However, relatively little is known about whether such exposure affects culturally familiar and Western foods in the same manner. This question can be investigated effectively using EEG because event-related potentials provide temporally precise information about different stages of food-cue processing. In addition, multivariate analyses of EEG activity can reveal distributed patterns of neural information that may not be apparent from individual ERP components alone.

Taken together, existing research highlights the importance of food cue exposure in shaping craving responses but leaves open the question of how cultural familiarity influence these processes. This gap is particularly important in societies undergoing rapid dietary transitions, where individuals are exposed simultaneously to traditional and Western foods. The present study addresses this gap by examining how multisensory exposure to indigenous Indian and Western foods influences craving responses in an Indian population. Using a cue-exposure paradigm, participants were exposed to images of culturally familiar Indian foods and Western food items representing sweet and savory categories. Behavioral responses were assessed using desire ratings, willingness-to-pay measures, and the Food Craving Questionnaire-State, while neural responses were recorded using a 64-channel EEG system. We hypothesized that culturally familiar indigenous foods would elicit stronger craving responses and higher subjective valuation than Western foods due to learned associations and cultural familiarity. Additionally, we explored how food type (sweet vs. savory) modulates responses to both Western and Indian foods. By integrating behavioral measures of craving with electrophysiological recordings, the present study aims to provide new insights into how cultural exposure, food type and multisensory exposure interact to shape food craving and eating behavior.

## 2 Materials and methods

### 2.1 Ethical statement

Written informed consent was obtained from all participants before the commencement of the experiment. The study was conducted in full compliance with the ethical principles outlined in the Declaration of Helsinki. The experimental protocol received formal approval from the Institute Ethics Committee of the institute (No: IISERK/IEC/2021/04), ensuring that all procedures adhered to established guidelines for research involving human participants.

### 2.2 Participants

A total of 54 participants participated in this study. The first experiment (IFC) was conducted with 28 participants (14 male and 14 female, ages 18–29, mean: 22.32, std: 2.48), of whom 14 were exposed to Indian sweet and 14 to Indian savory food items. In the second experiment (WFC), 26 participants (15 male and 11 female, ages: 18–29, mean: 22.42, std: 3.16) participated, of whom 14 were exposed to Western sweet and 12 to Western savory food items. All the participants were students or researchers from our institute. As the institute's student population comprises individuals from across India, it represents a diverse range of cultural backgrounds, thereby enhancing the generalizability of the findings to the broader Indian population. Participants were excluded if they were following any dietary restrictions (whether medically prescribed or self-imposed), had a history of eating disorders or chronic illnesses, were under 18 years of age, or had a Body Mass Index (BMI) indicating obesity (≥ 30) or underweight status (< 18). A participant was also excluded from the experiment if his/her time duration of refraining from eating before the experiment was either less than two or more than 6 h.

### 2.3 Procedure

The behavioral and corresponding EEG data were collected for 54 participants, 28 of whom were exposed to Indian food items and 26 of whom were exposed to Western food items. India is a culturally diverse country with a rich and varied cuisine. Hence for culturally familiar food items, food images were chosen very carefully. The participant pool included students and researchers from different regions of India with varying preferences for food depending on their culture. The culturally familiar stimulus set therefore consisted of popular and widely available Indian sweet and savory snack items that were expected to be familiar to participants from different regional backgrounds (Supplementary Figure S1).

A previous study (Konova et al., 2018) showed that irrespective of the number of stimulus types, category (sweet/savory) is the most significant predictor of the similarity between two Western food items. Accordingly, chocolate-based items were used as Western sweet stimuli, whereas chips were used as Western savory stimuli (Supplementary Figure S2). All food items used as stimuli were vegetarian. For the familiarity of the stimuli, we chose the food items that are available in the IISER Kolkata canteen to ensure stimulus familiarity across all participants. A pre-questionnaire survey was conducted in which students rated their preference for the food items included in the stimulus set on a scale of 1 to 10. Ratings of four sweet and four savory food images were obtained for IFC, and for WFC, five sweet images and five savory food images' ratings were obtained. Separate one-way repeated-measures ANOVAs were conducted for the eight IFC and ten WFC food images. The liking ratings were not significantly different in either experiment (IFC stimuli: *F* = 2.02, (*df*1, *df*2) = (7, 208), *p* = 0.0535; WFC stimuli: *F* = 0.9, (*df*1, *df*2) = (9, 240), *p* = 0.52). *Post-hoc* analysis revealed no significant pairwise difference between images for both IFC (Supplementary Table S1) and WFC (Supplementary Table S2). The survey ratings revealed no significant overall difference in participants' liking of different food stimuli, indicating that the subsequent analyses were unlikely to be influenced by a systematic baseline preference for a particular stimulus. It should be noted that the food images used in the survey were different from those employed as experimental stimuli, although they belonged to the same food categories. The stimuli set consists of both images generated by us and also obtained from internet sources under Creative Commons licenses. The stimuli set was pre-processed by cropping and resizing all images to 500 × 350 pixels. All images were standardized for low-level visual properties, including luminance, contrast, and saturation, prior to stimulus presentation. Participants' preferences for food were recorded previously, and since their liking did not differ significantly across stimuli, they were exposed to the foods randomly in both experiments. To enhance motivation for the sweet and savory food items, participants were required to refrain from eating or drinking (except water) for a minimum of 2 h before the experiment. The time of their last meal was recorded at the beginning of the experiment. All participants completed the Food Craving Questionnaire-State (FCQ-S) (Cepeda-Benito et al., 2000; Nijs et al., 2007) before starting and after completing the experiment. FCQ-S is a 15-item well-founded and definitive measure of state fluctuations in self-reported food craving. Each item is rated on a 5-point likert scale (1 to 5), and the responses are summed to generate a total state Food Craving score ranging from 15 to 75. High scores reflect strong food craving experience and low scores suggest weaker food craving experience.

#### 2.3.1 Experimental task

The task contains 15 blocks (2 practice + 3 pre-exposure + 10 post-exposure blocks). The first two blocks were for the participants to be habituated to the paradigm, so these two blocks were not considered for the analysis of the experiment. The three pre-exposure blocks worked as a baseline measure of their craving, and the post-exposure blocks were used to see the change in craving due to multisensory exposure. Additional post-exposure blocks were included to examine how craving changes across blocks. Each block consists of 20 trials (10 sweet + 10 savory), leading to a total of 260 trials. Each trial (Figure 1) presented a high-resolution color image of a food item for 1 s, followed by a green-colored desire rating scale displayed for 12 s or until a response was made, and then a deep blue bidding scale shown for the same duration as the desire scale. Both the scales were controlled through the mouse with a slider provided to help reach the desired response. The desire scale ranged from –100 to 100, where –100 stands for minimum desire and 100 indicates maximum desire for the food item. Participants were given a virtual 400 endowment for each block, with which they could bid for the food items. So the bidding scale was dynamic and it changed across trials. It ranges from 0 to the amount left for the rest of the trials (i.e., 400- amount spent till the trial) in a particular block. Within each trial, the order of the desire and bidding scales was randomized to prevent participants from anticipating which scale would appear next. Participants were clearly instructed to evaluate their current level of desire for the displayed food item, as well as to assign a subjective value using a virtual monetary bid. Trials were presented in a randomized sequence within each block; the same randomized sequence was used across participants. Each participant experienced multisensory food exposure for a maximum of 2 minutes after completion of three pre-exposure blocks and before 10 post-exposure blocks to induce craving. Participants were given a real food item (sweet or savory) and instructed to sense it by smelling and seeing it without having it. For IFC, the participants were given an Indian sweet or savory food item. In WFC, the participants were given a Western sweet or savory food item. The entire experimental procedure was monitored via a camera from outside the experiment room, specifically to ensure that participants refrained from eating during the multisensory exposure. All participants received 400 plus a bonus amount, which is calculated using a modified Becker-DeGroot-Marschak (BDM) mechanism (Becker et al., 1964), and a snack for their participation. In our experiment, one block was selected at random, and, within that block, for each trial, the proportion of the bid amount to the left amount was calculated. Then a random number was generated between 0 and 1. If that proportion exceeded the random number, the participants won an extra unit and if the proportion was less than that random number, then the participant won no extra unit. So a participant received a total of 400 + number of units won × 10.

**Figure 1:**
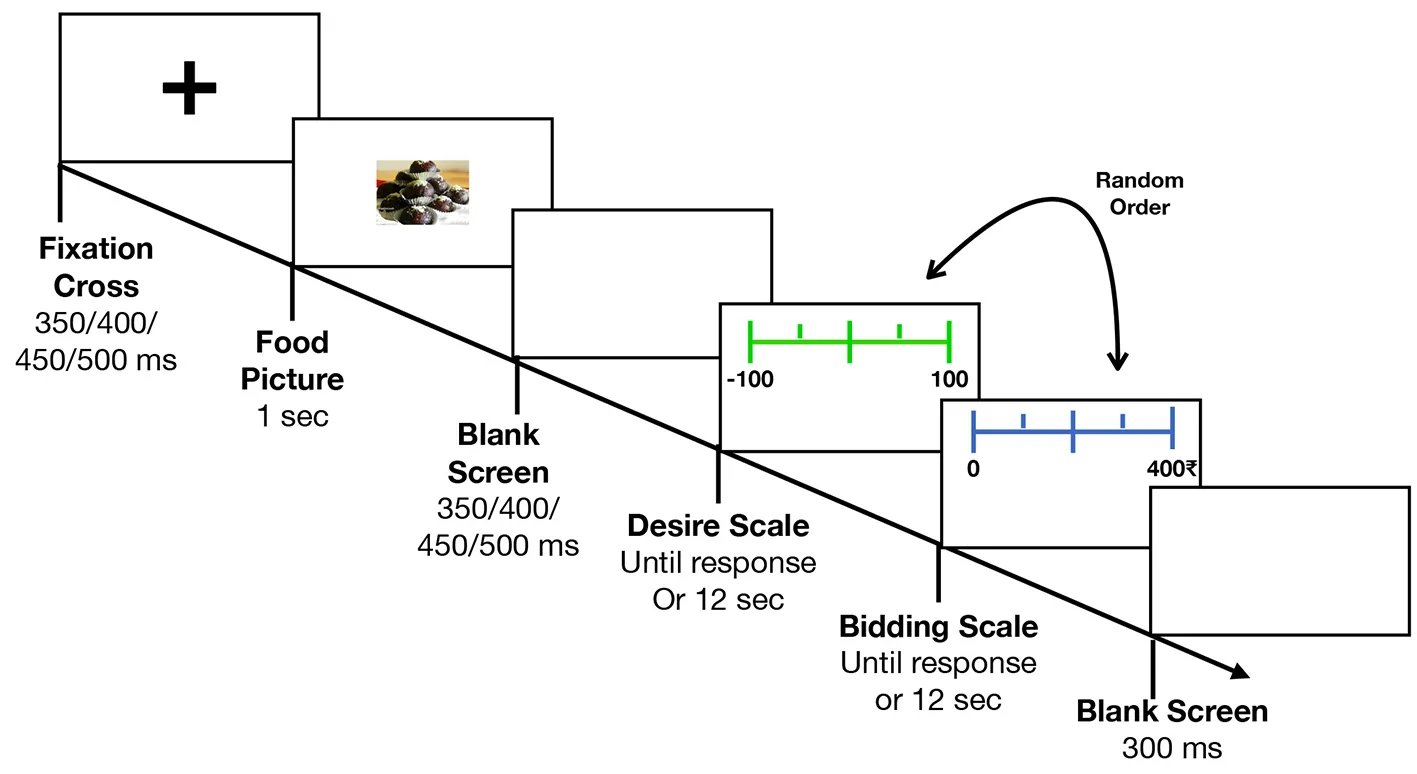
Paradigm of the experiment. One trial will start with a fixation cross with a jitter time window (350–500 ms). Then the food stimuli will be there for 1 s followed by a blank screen. After that desire and bidding scale will be there in random order followed by another blank for 300 ms.

### 2.4 Neural data acquisition and pre-processing

EEG data were recorded using a 64-channel active shielded electrode cap arranged according to the international 10/20 system. Recordings were made at a sampling rate of 512 Hz using two linked Nexus-32 bioamplifiers. Trial segmentation was performed based on trigger information, and all segments were pre-processed using the EEGLAB toolbox (Delorme and Makeig, 2004). Trials were epoched with stimulus onset as 0 ms, with a −200 ms baseline and 600 ms post-stimulus window. The EEG signals were band-pass filtered between 0.01 and 50 Hz, baseline-corrected, and re-referenced to the average of all electrodes. Trials containing ocular artifacts such as eye blinks or movements were identified using bipolar electro-oculogram (EOG) and visual inspection. Additionally, trials with amplitudes exceeding ±100 μV were excluded (Nijs et al., 2010). Following artifact rejection and outlier removal, an average of 119.22 ± 8.08 trials out of 130 trials per participant were retained for analysis for each food category (sweet and savory). After the trial rejection, Independent Component Analysis (ICA) was performed and components having artifacts were removed using Multiple Artifact Rejection Algorithm (MARA) (Winkler et al., 2011), a plug-in in EEGLAB. Noisy electrodes were removed before ICA and the data for the same electrodes were interpolated using spherical interpolation based on the spatial locations of the surrounding electrodes after ICA, as implemented in EEGLAB.

### 2.5 Behavioral data analysis techniques

#### 2.5.1 Preliminary analyses

Participants' subjective hunger ratings immediately before the experiment were also recorded. To examine whether pre-experiment hunger was associated with changes in bid values from pre- to post-exposure, we fitted a linear mixed-effects model with change in bid as the response variable, hunger rating as a continuous predictor, and culture and food type as categorical predictors. Although only participants with BMI values between 18 and 30 were included, we additionally examined whether BMI was associated with changes in bid values using a linear mixed-effects model with change in bid as the response, BMI as a continuous predictor, and culture and food type as categorical predictors. To illustrate the variability in bid values across participants, boxplots (see Supplementary Figure S3) were constructed for each subject. The plots show substantial dispersion in bid values, indicating considerable between-subject variability. The participants' FCQ-S values before and after the experiment were used to measure the induced craving. To compare the FCQ-S values between before and after conditions of the neuroeconomic decision-making task, the total FCQ-S value is computed for each of the participants by adding the response values (from 1 to 5) for all 15 questions. A paired t-test was then conducted to compare the before and after conditions for both types of food exposure (sweet and savory) across both experiments (IFC and WFC). To examine whether multisensory exposure influenced participants' craving, we conducted paired t-tests comparing bid and desire ratings before and after exposure for each food type and culture. All *t*-tests were conducted using one-tailed tests, as our a priori hypothesis was that craving would increase following the multisensory exposure. To account for multiple comparisons across the paired t-tests, p-values were adjusted using the Benjamini–Hochberg (BH) false discovery rate (FDR) procedure within each prespecified family of related hypotheses. Because the IFC and WFC studies involved separate participant samples and addressed the effects of exposure within distinct food cultures, multiple correction was performed separately for the two Experiments. Also, the group of participants who were exposed to sweet items as multi-sensory exposure are different from those who were exposed to savory items, multiple comparison tests are done independently for those groups as well. The difference in bid and desire values are considered to be a single family as they are similar behavioral measures and multiple comparisons are performed for them.

Given the relatively small sample size, we additionally conducted a sensitivity analysis to determine the minimum within-subject effect size detectable with 80% power. The analysis was performed in R using the pwr.t.test function from the pwr package. At the nominal α = 0.05 level, a one-tailed paired *t*-test with *n* = 14 provides 80% power to detect an effect size of approximately *d*_*z*_ = 0.70, whereas for *n* = 12, an effect size of approximately *d*_*z*_ = 0.76 is required. These calculations characterize the sensitivity of the individual paired comparisons and do not incorporate the subsequent BH adjustment for multiple testing. Thus, the available sample size provided reasonable sensitivity to relatively large within-subject effects but limited sensitivity to small or moderate effects.

Bayesian paired-samples t-tests were conducted using the ttestBF() function from the BayesFactor package in R. Bayes factors (*BF*_10_) quantified the evidence for the alternative hypothesis relative to the null hypothesis. The analyses employed the default Jeffreys–Zellner–Siow (JZS) prior with a Cauchy prior distribution on the standardized effect size (*r* = 0.707) (Rouder et al., 2009). Bayes factor (*BF*_10_ values were not reported when they required approximation using the Bayesian Information Criterion (BIC). Bayes factors were interpreted using conventional criteria, where *BF*_10_ >3, >10, and >30 indicated moderate, strong, and very strong evidence for the alternative hypothesis, respectively, whereas values below 1 favored the null hypothesis.

#### 2.5.2 Computational model

To examine how multisensory exposure modulates bid values across food types and cultural contexts, we fitted a linear mixed-effects model with autocorrelated residuals. This framework allowed us to simultaneously assess the effects of multiple experimental factors while accounting for subject-level variability and serial dependence across trials.

Bid reaction time was included as a covariate in the model. However, reaction time for a given trial is itself influenced by several factors, including the desire value associated with the trial, exposure condition (pre- vs. post-exposure), food type, and culture. Consequently, the observed reaction time reflects variation attributable both to these factors and to trial-specific processes. To isolate the unique contribution of reaction time, we first removed the effects of these factors from reaction time and used the resulting residualized reaction time as a covariate in the model. For that purpose, we adopted a two-stage modeling approach. First, the logarithm of bid reaction time (LBRT) was modeled as a function of desire rating, time (pre vs. post), exposed food type, and culture. To account for between-subject heterogeneity in baseline bidding behavior, subject was included as a random intercept. The residuals from this model were then extracted to obtain reaction-time variability independent of these covariates. In the second stage, after removing the 0 and 1 bid trials from the data (~7.5% of the total trials), $\log\left(\frac{\text{bid}}{1-\text{bid}}\right)$ value was modeled as a linear function of the residuals along with the same set of covariates used in modeling LBRT. This transformation was applied because the bid values, defined as the ratio of the bidden amount to the remaining amount, are bounded between 0 and 1. The transformation maps these bounded values onto the real line, allowing us to use a linear model. All experimental factors were included as fixed effects. Subject was included as a random intercept to account for between-subject variability, together with participant-specific random slopes for desire and residualized reaction time (RoRT). Additionally, to accommodate serial dependence across repeated trials within each subject, an autoregressive AR(1) correlation structure was specified for the residuals. If we fit the bid model without the AR(1) process, then there is autocorrelation between residuals (Supplementary Figure S6).

Accordingly, two statistical models were specified: one for bid reaction time and one for transformed bid value. We introduce the following notation to represent the statistical models.


$$\begin{array}{l} \;y_{ij}=\log\left(\frac{\text{bid}}{1-\text{bid}}\right)\;\text{value for }i^{th\;}\text{ trial and }j^{th}\;\text{participant}\\ \;D_{ij}=\text{desire rating for}i^{th}\text{ trial and }j^{th}\text{ participant}\\ LBRT_{ij}=\text{logarithm of bid reaction time for}{\;i}^{th\;}\text{trial and }j^{th\;}\\ \;\text{participant} \end{array}$$

$$\begin{array}{l} T_{ij}=\text{time (pre or post) for }i^{th\;}\;\text{trial and }j^{th\;}\text{participant}\\ F_{j}=\text{exposed food type for}{\;j}^{th\;}\text{participant}\\ C_{j}=\text{culture for}{\;j}^{th\;}\text{participant} \end{array}$$

The bid reaction time model is provided as


$$\begin{array}{l} LBRT_{ij}=\alpha_{0}+\alpha_{1}D_{ij}+\alpha_{2}T_{ij}+\alpha_{3}F_{j}+\alpha_{4}C_{j}+a_{0j}+\eta_{ij}, \end{array}$$ (1)

where α_0_ is the intercept, α_*k*_, *k* = 1, …, 4, are fixed effects coefficients, *a*_0*j*_ is subject specific random intercept, and $\eta_{ij}\sim N\left(0,\sigma_{\eta }^{2}\right)$, a random noise. Here we have not taken any interactions as they are not significant (see Supplementary Table S3 in Supplementary B). As this model was not the primary focus of our analysis, we deliberately avoided overparameterization. From this model, the residual of the reaction time (RoRT) is calculated as


$$R_{ij}=LBRT_{ij}-{\hat{LBRT}}_{ij},$$

where ${\hat{LBRT}}_{ij}$ is the fitted log reaction value based on the model (Equation 1). We then propose the following model for the transformed bid value below.


$$\begin{array}{l} y_{ij}=\beta_{0}+\beta_{1}D_{ij}+\beta_{2}R_{ij}+\beta_{3}T_{ij}+\beta_{4}F_{j}+\beta_{5}C_{j}+\beta_{6}\left(T_{ij}\times F_{j}\right)\\ \;+\beta_{7}\left(T_{ij}\times C_{j}\right)+\beta_{8}\left(F_{j}\times C_{j}\right)+\beta_{9}\left(T_{ij}\times F_{j}\times C_{j}\right)+b_{0j}\\ \;+b_{1j}D_{ij}+b_{2j}R_{ij}+\epsilon_{ij} \end{array}$$ (2)

where *T*_*ij*_×*F*_*j*_, *T*_*ij*_×*C*_*j*_, *F*_*j*_×*C*_*j*_, *T*_*ij*_×*F*_*j*_×*C*_*j*_ represents interaction between time and food type (IbTF), interaction between time and culture (IbTC), interaction between food type and culture (IbFC), interaction between time, food type and culture (IbTFC), β_0_ is the grand intercept, β_1_, …, β_9_ are fixed effect coefficients, and *b*_0*j*_ is participant specific random intercept. We also included random slopes for desire and RoRT with respect to the participants. Thus, ${\left(b_{0j},b_{1j},b_{2j}\right)}^{\prime }\sim N_{3}\left(\text{0},\Sigma \right)$.

The model included a participant-specific random intercept, together with participant-specific random slopes for desire and RoRT, to account for heterogeneity in both baseline responses and the associations of these continuous within-participant covariates with the outcome. Random slopes were not specified for food type or culture because these variables are between-participant factors and therefore do not vary within a participant. Consequently, their participant-specific slopes cannot be separately identified from the participant-specific intercept.

Although time (pre- vs. post-exposure) varies within participants and could, in principle, be assigned a random slope, only two repeated time points were available for each participant and the participant-level sample sizes were relatively small. Given that the model already contains a participant-specific random intercept and random slopes for desire and RoRT, introducing an additional random slope for time would require estimation of further variance–covariance components from limited within-participant information. We therefore used a parsimonious random-effects structure in which the pre–post effect was modeled as a population-level fixed effect, while participant-specific heterogeneity was accommodated through the random intercept and the random slopes for desire and RoRT.

Since there is autocorrelation present between residuals of the trials (Supplementary Figure S6), we propose a AR(1) structure on the error ϵ_*ij*_ as


$$\epsilon_{ij}=\rho \epsilon_{i-1,j}+u_{i,j},|\rho |<1,u_{ij}\sim N\left(0,\sigma^{2}\right).$$

Statistical significance of fixed effects was assessed using F-tests obtained from analysis of variance (ANOVA). Given that both participants and stimuli were sampled from larger populations and that the model was estimated using trial-level observations, it is necessary to account for the possibility that a substantial proportion of the variance arises from between-participant heterogeneity rather than from trial-to-trial variation. To see the effect of this between participants compared to trial variation, we provide a caterpillar plots and compute the Intraclass Correlation Coefficient (ICC) (McGraw and Wong, 1996; Hox et al., 2017) for a quantitative assessment. Caterpillar plot is a plot of sorted random effects of the model, whose spread will show the variation among participants. ICC is the proportion of the total residual variation in bid that is attributable to stable differences between participants, after accounting for the fixed effects in the model. ICC is defined as $ICC=\frac{\sigma_{b}^{2}}{\sigma_{b}^{2}+\sigma_{\epsilon }^{2}}$, where $\sigma_{b}^{2}$ is the between-subject variance (for our case, random intercept variance) and $\sigma_{\epsilon }^{2}$ is the trial-level variance.

To see how bid changes across blocks after the multisensory exposure, we modeled the change in bid compared to pre-exposure using block number, food type, and culture as follows:


$$\begin{array}{l} \Delta bid_{ij}=\gamma_{0}+\gamma_{1}block_{ij}+\gamma_{2}F_{j}+\gamma_{3}C_{j}+c_{0j}+c_{1j}block_{ij}+\epsilon_{ij} \end{array}$$ (3)

where Δ*bid*_*ij*_ is the change in bid in the *i*^*th*^ block for *j*^*th*^ subject. Subject-specific random intercepts and random slopes for bid were incorporated into the model.

### 2.6 Neural data analysis techniques

#### 2.6.1 Univariate analysis

To see the effect of multisensory exposure on sweet and savory food items, we investigated P200, N200, and Late Positive Potential (LPP) for indigenous and Western food. P200 was measured in the frontal region using the electrode cluster F1, F2, F3, F4, AF3, AF4, Fz (Nijs et al., 2010) around 200 ms after the stimulus onset. N200 was measured in the parietal region using the electrode cluster P1, P2, P3, P4, Pz, PO3, PO4 (Chatterjee et al., 2023) around 200 ms after stimulus onset. To measure LPP we have considered centro-parietal electrode cluster CP1, CP2, CP3, CP4, P1, P2 (Carbine et al., 2017) in the 300 to 600 ms time window. We calculated the peak amplitudes around 200 ms by taking the time window 150–250 ms to identify the P200 (Plihal et al., 2001) and N200 (Jimenez et al., 2018) ERP components. For P200, we took the maximum amplitude in that time window and for N200, the minimum amplitude has been taken in that time window. For the LPP, the average amplitude within the 300–600 ms time window was used. To compare the ERP components between pre-exposure and post-exposure conditions, we did paired *t*-test for each food culture and each food item.

Partial correlations were computed to examine the relationships between behavioral responses (desire and bid ratings) and ERP components (P200, N200, LPP) across all food categories under pre-exposure and post-exposure conditions. Partial correlation was calculated between two variables, controlling for the other variables. Following (Cohen, 2013; Kelley and Preacher, 2012), we considered correlations as moderate for *r*>0.24 and large for *r*>0.3.

#### 2.6.2 Multivariate analysis

In the univariate EEG analysis, electrodes were grouped into predefined clusters and averaged to examine ERP components, which limited the exploitation of the spatial structure of the data. To overcome this limitation, we employed multivariate pattern analysis (MVPA), which leverages inter-electrode dependencies to capture distributed spatial patterns in multichannel EEG signals.

MVPA was preceded by a recently proposed data-driven channel selection procedure based on Functional Data Analysis (FDA) (Das et al., 2023). Unlike the univariate approach, which relied on electrode groupings informed by prior literature, the FDA-based method is data-driven and does not rely on predefined anatomical electrode clusters. The procedure consists of two stages: (i) selection of electrodes exhibiting the highest neural activity, and (ii) ranking of these electrodes according to their discriminatory power between classes. Ranked clusters were then constructed using both discriminatory rankings and functional correlations among electrodes. A fixed number of electrodes was selected from each cluster to maximize discriminative information while minimizing redundancy.

Classification was performed between pre- and post-exposure conditions for both food and cultural stimuli. For MVPA, first three blocks of post-exposure were chosen with the three pre-exposure blocks to obtain balanced numbers of pre- and post-exposure trials. Single-trial EEG signals were classified using a linear support vector machine (SVM). A 10-fold cross-validation scheme was implemented independently for each participant, with channel selection performed exclusively on the training data in each fold to prevent information leakage. Performance was quantified as mean classification Area Under Curve (AUC) averaged across participants. To assess whether the observed AUC was significantly greater than chance, we performed a label-permutation test (Combrisson and Jerbi, 2015; Stelzer et al., 2013). For each participant, the class labels were randomly permuted 50 times, and the classification analysis was repeated for each permutation using 10-fold cross-validation, yielding 50 null AUC values per participant. To obtain a group-level null distribution, 10^5^ bootstrap resamples were subsequently generated from participant-specific permutation-derived null distributions. For each resample, one permutation-derived AUC was sampled with replacement for each participant, and the resulting AUCs were averaged across participants. This procedure yielded a null distribution of (10^5^) group-level mean AUCs. The one-sided permutation (*p*)-value was calculated as the proportion of values in this null distribution that were greater than or equal to the observed mean AUC.

To investigate the effect of early and late processing, we separately classified the EEG signals from the two time windows. Classification was performed in the time window 0-300 ms and 300-600 ms and AUC values are reported. The same permutation test procedure was used to determine whether classification performance exceeded chance.

## 3 Results

### 3.1 Behavioral data analysis

#### 3.1.1 Preliminary analyses

To examine whether participants' immediate pre-experiment hunger ratings were associated with changes in bid values, we fitted a linear mixed-effects model including hunger as a continuous predictor and culture and food type as categorical predictors, together with their interactions. Fitted model results are given in Supplementary Table S4. The ANOVA of the fitted mixed-effects model showed no significant overall effect of hunger on the change in bid values (*F*_1, 46_ = 0.67, *p* = 0.41; Supplementary Table S5). Moreover, none of the interaction terms involving hunger were significant. Similarly, the model results fitted to see if BMI has any effect on the change in bid are given in Supplementary Table S6. The ANOVA of this fitted model showed no significant overall effect of BMI on the change in bid (*F*_1, 46_ = 0.001, *p* = 0.96; Supplementary Table S7). We conducted paired-sample t-tests to compare FCQ-S scores measured before and after the experiment. Across both cultural categories and food types, FCQ-S scores increased significantly following exposure, indicating an elevation in state food craving induced by multisensory stimulation. Specifically, for IFC, FCQ-S scores increased for both sweet (*t*_13_ = 3.10, *p* = 0.0042, effect size = 0.83, *BF* = 6.57) and savory (*t*_13_ = 3.53, *p* = 0.0019, effect size = 0.94, *BF* = 12.86) foods. A similar pattern was observed for WFC, with significant post-exposure increases for both sweet (*t*_13_ = 2.28, *p* = 0.02, effect size = 0.61, *BF* = 1.86) and savory (*t*_11_ = 4.18, *p* = 7.63 × 10^−4^, effect size = 1.21, *BF* = 28.04) items.

We additionally performed paired-sample *t*-tests to compare bid and desire ratings between pre- and post-exposure conditions. For Indian foods, both bid (sweet: *t*_13_ = 2.89, *p* = 0.0063, *p*_*BH*_ = 0.0066, effect size= 0.77, *BF* = 4.69; savory: *t*_13_ = 2.49, *p* = 0.013, *p*_*BH*_ = 0.0198, effect size=0.67, *BF* = 2.54) and desire ratings (sweet: *t*_13_ = 2.86, *p* = 0.0066, *p*_*BH*_ = 0.0066, effect size=0.76, *BF* = 4.5; savory: *t*_13_ = 2.29, *p* = 0.0198, *p*_*BH*_ = 0.0198, effect size=0.61, *BF* = 1.89) showed significant post-exposure increases. Thus, the exploratory data analysis indicates that there may be a significant increase in bid and desire values from pre-exposure to post-exposure for the Indian food stimuli. On the contrary, the evidence for changes in Western foods (Experiment 2) was weaker. For Western sweet foods, desire ratings increased following exposure (*t*_13_ = 2.10, *p* = 0.0279, *p*_BH_ = 0.047, effect size = 0.56, *BF* = 1.45), although the corresponding Bayes factor indicated only weak evidence for this effect. A similar pattern was observed for bid values for Western sweet foods (*t*_13_ = 1.81, *p* = 0.047, *p*_BH_ = 0.047, effect size = 0.48, *BF* = 0.98), for which the Bayes factor provided little evidence favoring either hypothesis. No significant changes were observed for Western savory foods.

#### 3.1.2 Computational model

Model parameters for both Models Equations 1, 2 were estimated using restricted maximum likelihood (REML) as implemented in the nlme package in R. The fixed-effect estimates for the LBRT model are given in Table 1 and the bid value model are presented in Table 2.

**Table 1:** LBRT model fit results.

Coefficients of	Estimate	DF	*t*-value	*p*-value
Intercept (α_0_)	7.734	6230	118.60	< 0.001
Desire (α_1_)	0.053	6230	7.35	< 0.001
Time (α_2_)	–0.151	6230	–11.88	< 0.001
Food type (α_3_)	0.098	51	1.31	0.198
Culture (α_4_)	0.064	51	0.84	0.404

**Table 2:** Results of linear mixed effect model fit on bid values.

Coefficients of	Estimate	t-value	DF	*p*-value
Intercept (β_0_)	−1.9683	−10.87	6,226	< 0.001
Desire (β_1_)	0.7815	9.57	6,226	< 0.001
RoRT (β_2_)	−0.1057	−2.48	6,226	0.0133
Time (β_3_)	0.1835	3.20	6,226	0.0014
Food type (β_4_)	0.1278	0.50	50	0.6199
Culture (β_5_)	−0.3901	−1.50	50	0.1401
IbTF (β_6_)	−0.0820	−0.99	6,226	0.3212
IbTC (β_7_)	−0.1759	−2.15	6,226	0.0317
IbFC (β_8_)	0.5513	1.47	50	0.1467
IbTFC (β_9_)	0.0014	0.01	6,226	0.9908

Desire has a significant positive effect on LBRT (α_1_ = 0.05, *t* = 7.35, df = 6230, *p* < 0.001), showing higher desire will require more time to bid. But time has a negative effect (α_2_ = −0.15, *t* = −11.88, *df* = 6230, *p* < 0.001) on LBRT, indicating that after exposure, they needed less time to bid. Food type and culture have no significant main effect on LBRT. The marginal adjusted *R*^2^, which quantifies the proportion of variance explained by the fixed effects in the model, is 0.039. And the conditional adjusted *R*^2^, which gives the proportion of variance explained by both fixed and random effects, is 0.32.

Desire ratings showed a positive association with bid values (β_1_ = 0.78, *t* = 9.57, *df* = 6226, *p* < 0.001), indicating that stronger subjective desire was robustly related to higher monetary bids. The residualized reaction time of bidding (RoRT) was significantly negatively associated with bid values (β_2_ = −0.105, *t* = −2.48, *df* = 6226, *p* = 0.013), suggesting that slower-than-expected responses were linked to lower bids. Time (pre vs. post exposure) also had a significant positive effect (β_3_ = 0.183, *t* = 3.2, *df* = 6226, *p* = 0.0014), reflecting an overall increase in bid values following multisensory exposure.

Neither food type (*t* = 0.5, *df* = 50, *p* = 0.62) nor culture (*t* = −1.5, *df* = 50, *p* = 0.14) showed significant main effects. The interaction between time and culture is significant (β_7_ = −0.17, *t* = −2.15, *df* = 6226, *p* = 0.031), suggesting a moderation of the exposure effect by cultural background. All other interaction terms were non-significant (*p*'s > 0.14).

The corresponding analysis of variance (ANOVA) table is provided in Table 3. Consistent with the parameter estimates, significant main effects were observed for desire ratings [*F* = 100.15, (*df*1, *df*2) = (1, 6226), *p* < 0.0001], bid reaction time residual [*F* = 6.16, (*df*1, *df*2) = (1, 6226), *p* = 0.013], and time [*F* = 4.75, (*df*1, *df*2) = (1, 6226), *p* = 0.02]. There were no significant main effects of food type [*F* = 3.19, (*df*1, *df*2) = (1, 50), *p* = 0.08] or culture [*F* = 1.92, (*df*1, *df*2) = (1, 50), *p* = 0.17].

**Table 3:** ANOVA results of the model fitted on bid values.

Variable	*F*-value	DoF	*p*-value
Intercept	442.49	(1, 6,226)	**< 0.0001**
Desire	100.15	(1, 6,226)	**< 0.0001**
RoRT	6.16	(1, 6,226)	**0.0131**
Time	4.75	(1, 6,226)	**0.0294**
Food type	3.19	(1, 50)	0.0801
Culture	1.92	(1, 50)	0.1718
IbTF	1.48	(1, 6,226)	0.2231
IbTC	8.30	(1, 6,226)	**0.0040**
IbFC	2.31	(1, 50)	0.1347
IbTFC	0.00	(1, 6,226)	0.9908

The bold *p*-values indicate that they are less than 0.05.

Importantly, the interaction between time and culture was statistically significant (*F* = 8.30, (*df*1, *df*2) = (1, 6226), *p* = 0.004), indicating that the effect of multisensory exposure on bid values differed across cultural groups. Although there was no overall difference in bid values between cultures, the magnitude of change from pre- to post-exposure varied by culture. *Post-hoc* comparisons revealed a significant increase in bid values from pre- to post-exposure for the Indian culture group (*t* = −3.45, *df* = 6226, *p* = 0.0006), whereas no significant pre–post difference was observed for the Western culture group (*t* = 0.74, *df* = 6226, *p* = 0.46). All *post-hoc* comparisons were adjusted for multiple testing using the Bonferroni correction to control the family-wise error rate and reduce the likelihood of Type-I errors arising from multiple statistical comparisons. The marginal adjusted *R*^2^ and conditional adjusted *R*^2^ for this bid model are 0.25 and 0.533, respectively. The ICC for this model is 0.5867. So approximately 58.67% of the total variance of bid is attributable to differences between subjects, suggesting that the results were not solely driven by the large number of trial-level observations. The caterpillar plots (Supplementary Figure S4) indicate substantial variability in bid values across subjects. We performed a residual analysis to assess the adequacy of the fitted model. The Q–Q plot (Supplementary Figure S5A), studentized residual plot (Supplementary Figure S5B), and ACF plot of the residuals (Supplementary Figure S5C) are presented. The Q–Q plot indicates some departure from normality, particularly in the upper tail, suggesting mild positive skewness. However, the studentized residual plot shows no clear evidence of heteroscedasticity, and the ACF plot indicates no substantial residual autocorrelation.

The interaction plot (Figure 2) further illustrates that bids for Indian foods increased more substantially following multisensory exposure compared to Western foods, supporting the interpretation that the exposure effect was culturally moderated. Model Equation 3, which was fitted to assess the effect of block, indicated no significant trend in change of bid values across blocks, as the block coefficient, γ_1_, was not statistically significant (see Supplementary Table S13).

**Figure 2:**
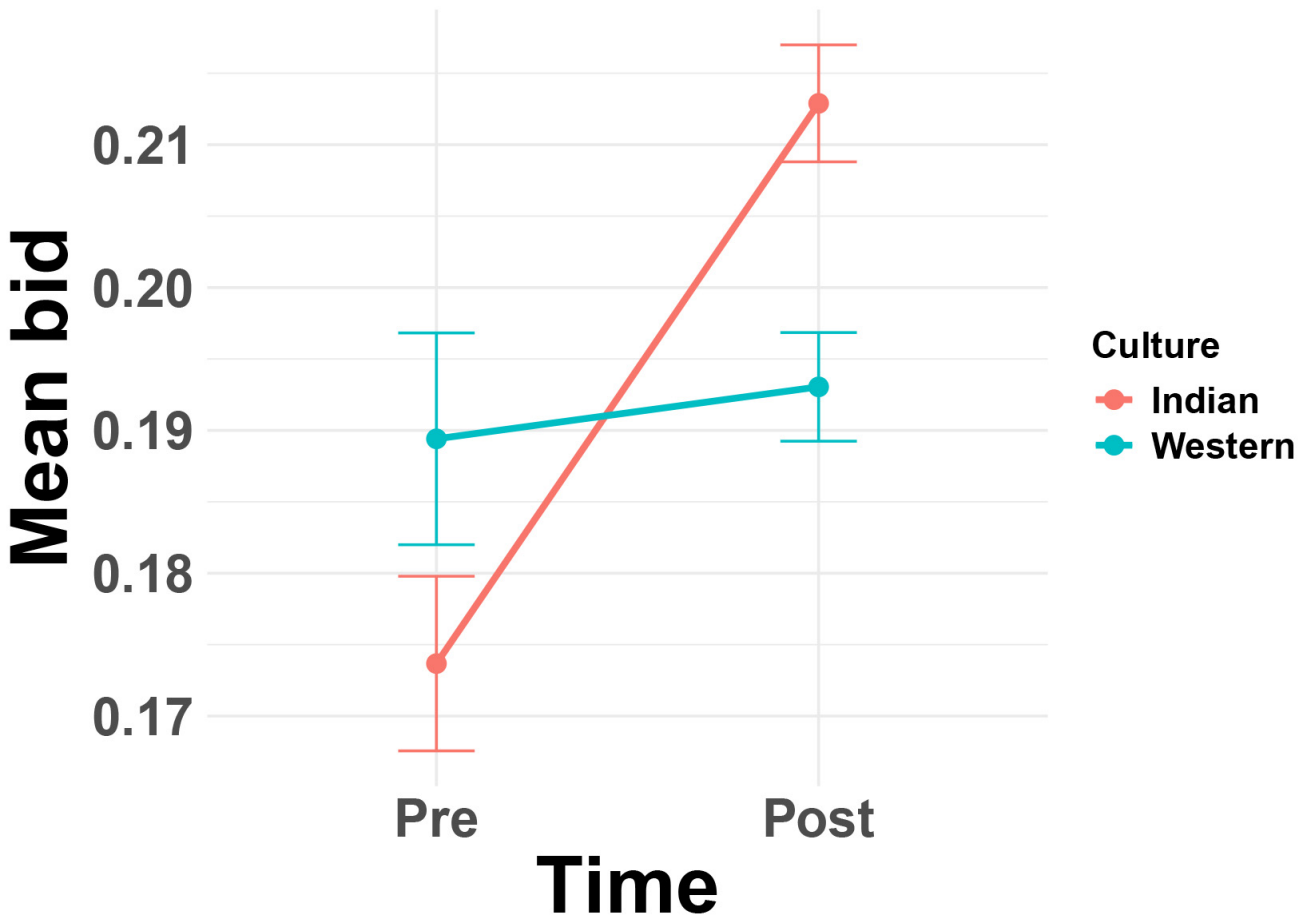
Interaction plot between time (pre and post) and culture. The vertical lines represent the standard error. From this plot, it is visible that the multisensory exposure has more effect for Indian foods compared to Western foods.

### 3.2 Univariate EEG results

We examined the grand-average waveforms to investigate differences in neural responses between the pre- and post-exposure conditions. In particular, we focused on the P200, N200, and Late Positive Potential (LPP) event-related potential (ERP) components. P200 and N200 peaks were measured in the time window 150–250 ms and LPP was measured in the time window 300–600 ms. Topoplots of the difference waveform of post- and pre-exposure of all food categories in those time windows are given in Supplementary Figure S7.

#### 3.2.1 P200

We have observed a positive-going peak around 200 ms after the stimulus onset in the frontal region for all food cultures and all food types (see Figure 3). A significant increase in P200 peak amplitude was found in post-exposure condition for Indian sweet food items only (*t* = 1.95, *df* = 13, *p* = 0.0364, effect size = 0.52, *BF* = 1.18). There is no significant increase in the P200 peak for any other food items.

**Figure 3:**
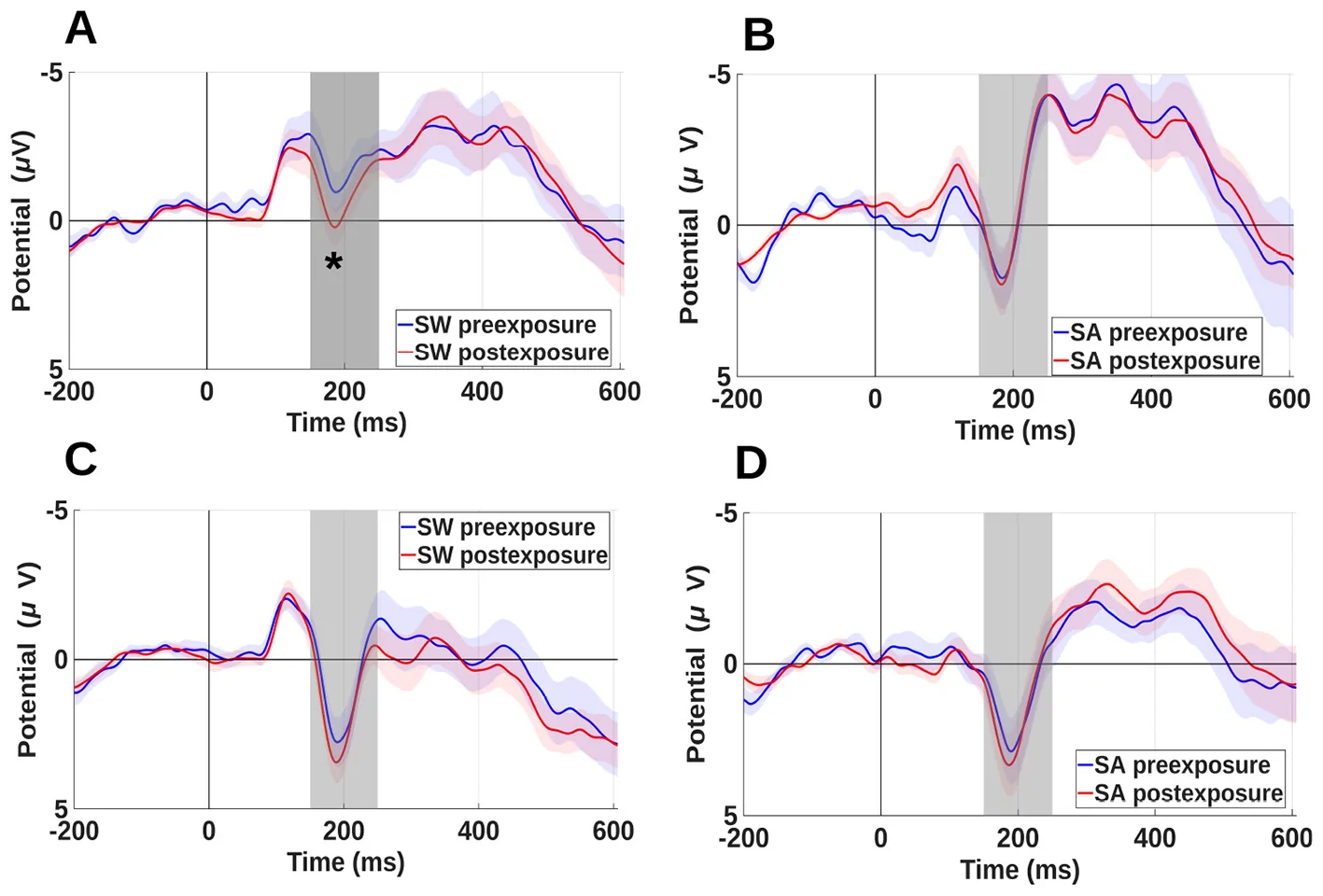
Event-related potential after 200 ms of stimulus onset in the frontal region (F1, F2, F3, F4, Fz, AF3, AF4) (P200). The figure shows EEG activity from 200 ms before to 600 ms after stimulus onset. The blue and red ones are pre- and post-exposure ERPs, respectively. **(A)** Indian sweet exposure; **(B)** Indian savory exposure; **(C)** Western sweet exposure; **(D)** Western savory exposure. The gray shaded regions indicate the time window when the positive-going peak is identified. The red and blue shaded regions around the ERPs indicate the standard error. The * indicates that the pre- vs. post-exposure *t*-test was significant for that food type.

#### 3.2.2 N200

We have observed a negative-going peak around 200 ms after the stimulus onset in the parietal region for all food cultures and all food types (Figure 4). Here also, a significant increase in N200 peak amplitude was observed for Indian sweet food items only (*t* = −2.06, *df* = 13, *p* = 0.0301, effect size = 0.55, *BF* = 1.36), and for any other food items we did not obtain any significant difference.

**Figure 4:**
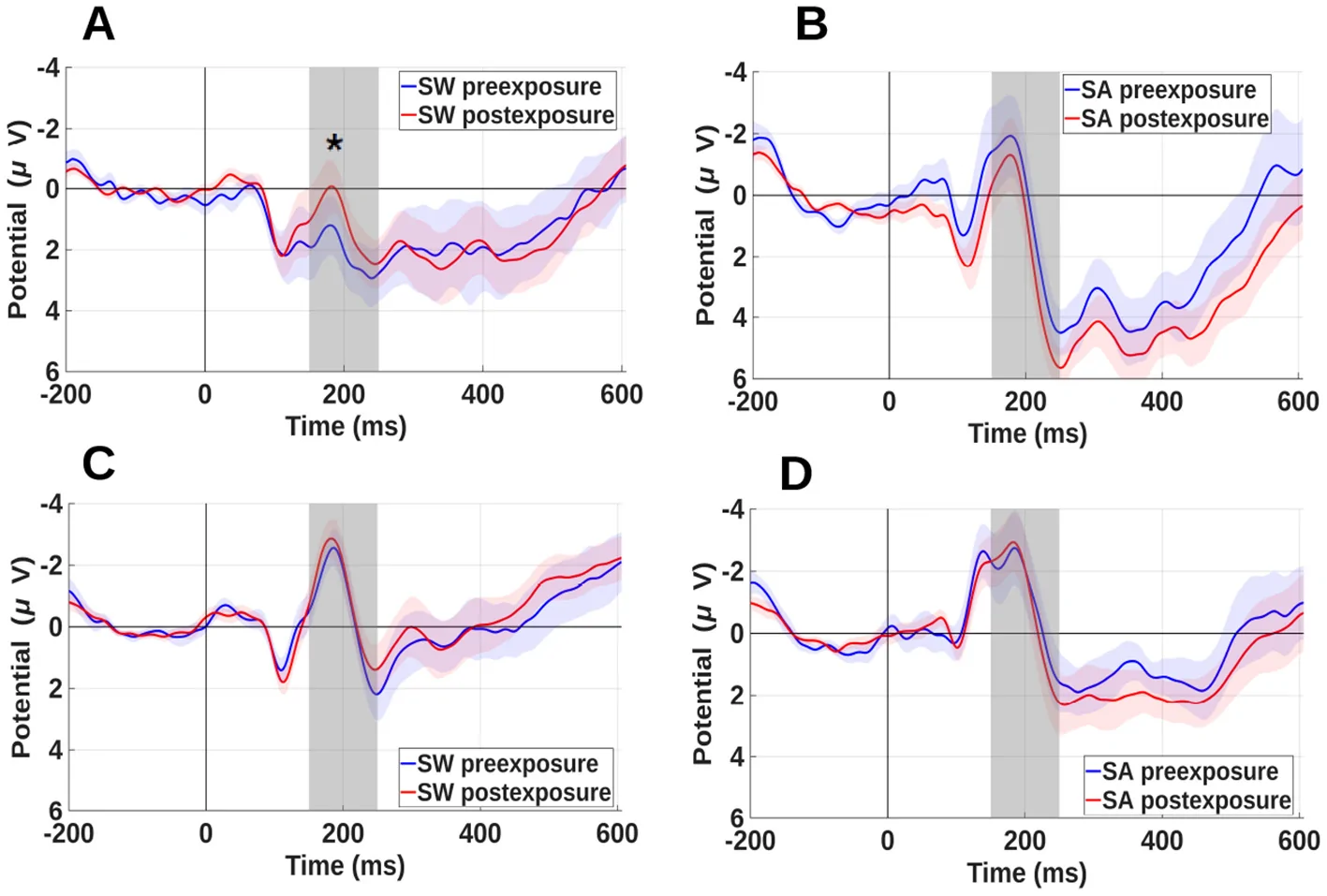
Event-related potential after 200 ms of stimulus onset in the parietal region (P1, P2, P3, P4, Pz, PO3, PO4) (N200). The figure shows EEG activity from 200 ms before to 600 ms after stimulus onset. The blue and red ones are pre- and post-exposure ERPs, respectively. **(A)** Indian sweet exposure; **(B)** Indian savory exposure; **(C)** Western sweet exposure; **(D)** Western savory exposure. The gray shaded regions indicate the time window when the negative-going peak is identified. The red and blue shaded regions around the ERPs indicate the standard error. The * indicates that the pre- vs. post-exposure *t*-test was significant for that food type.

#### 3.2.3 LPP

A sustained positive-going potential was observed over the centro-parietal region approximately 300–600 ms after stimulus onset across all food categories (Figure 5), consistent with the temporal and spatial characteristics of the late positive potential (LPP). LPP amplitudes were larger in the post-exposure than in the pre-exposure condition for Indian savory food items (*t*_13_ = 1.97, *p* = 0.0354, effect size = 0.52, *BF* = 1.21) and Western savory food items (*t*_11_ = 2.01, *p* = 0.0347, effect size = 0.58, *BF* = 1.29). No significant differences in LPP amplitude between the pre- and post-exposure conditions were observed for the sweet food items.

**Figure 5:**
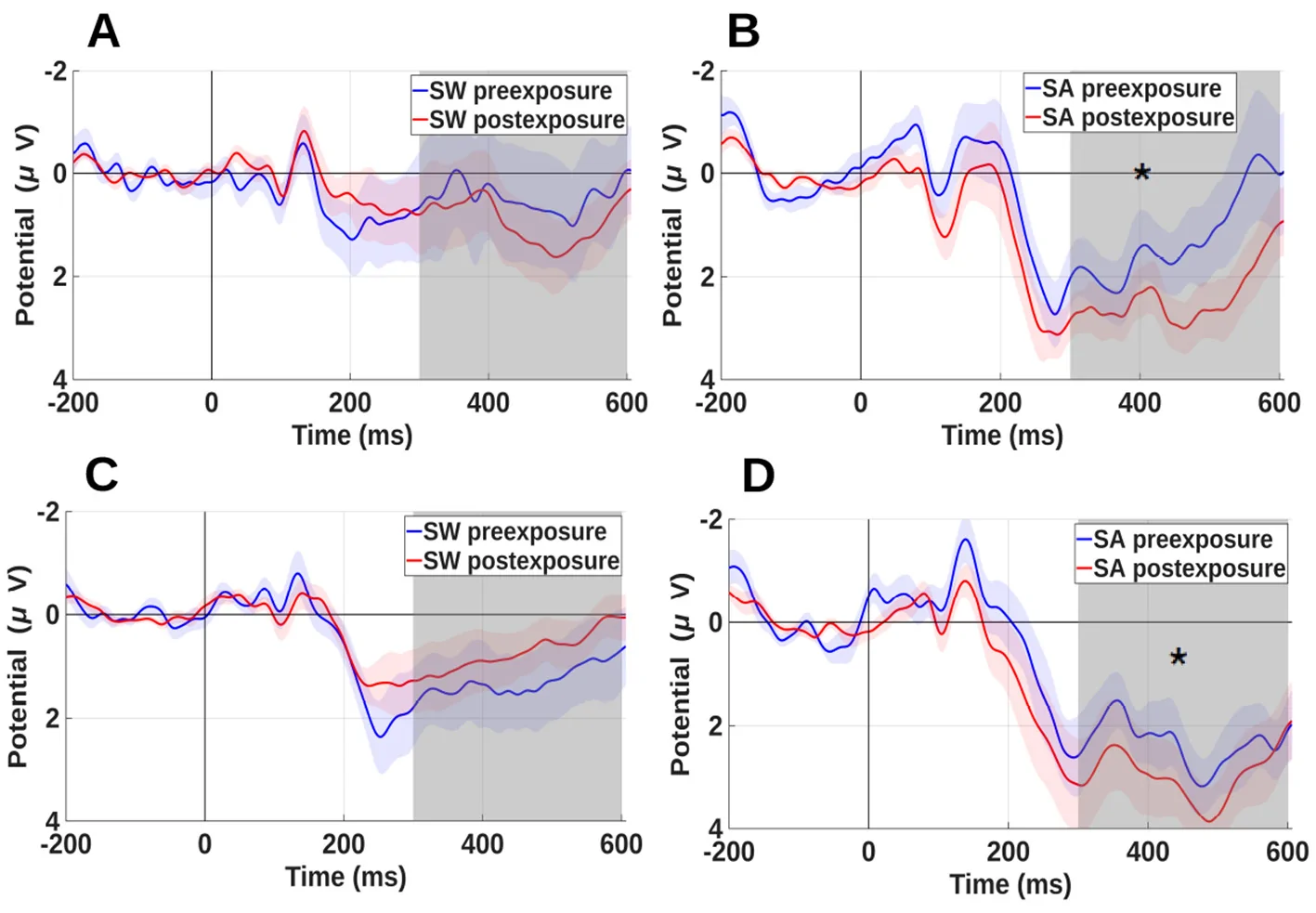
Event-related potential between 300 ms to 600 ms after stimulus onset in the centro-parietal region (CP1, CP2, CP3, CP4, CPz, P1, P2) (LPP). The figure shows EEG activity from 200 ms before to 600 ms after stimulus onset. The blue and red ones are pre- and post-exposure ERPs, respectively. **(A)** Indian sweet exposure; **(B)** Indian savory exposure; **(C)** Western sweet exposure; **(D)** Western savory exposure. The gray shaded regions indicate the time window where the late positive potential is identified. The red and blue shaded regions around the ERPs indicate the standard error. The * indicates that the pre- vs. post-exposure *t*-test was significant for that food type.

### 3.3 Effect of gender

To check the gender effect on food type and culture, we did 3-way factorial ANOVA on the difference of behavioral measures (desire and bid) and difference of ERP measures (P200, N200, and LPP) between post- and pre-exposure conditions separately. The results are reported in Supplementary Tables S8–S12 in Supplementary material E. For bid only, food type and gender has significant interaction (Supplementary Table S9). *Post-hoc* test shows that males' increment of bid after post-exposure is significantly better than females (*t* = –3.107, df=46, *p* = 0.0032, BF = 13.96) for savory food exposure only. Other measures (desire, P200, N200, LPP) have no effect of gender.

### 3.4 Multivariate pattern analysis

Following channel selection (using Das et al., 2023), classification between pre- and post-exposure conditions was performed using a support vector machine (SVM) with EEG data extracted from stimulus onset to 600 ms post-stimulus, and the resulting AUCs are shown in Figure 6. Permutation test shows that all AUCs are significantly greater than chance (Supplementary Figure S8). Classification AUC was higher for Indian sweet (AUC = 0.6027, se = 0.03, *p* < 0.0001, effect size = 0.85, BF = 36.29) and Indian savory (AUC = 0.6061, se = 0.037, *p* < 0.0001, effect size = 0.76, BF = 11.45) food items compared to Western sweet (AUC = 0.5691, se = 0.032, *p* = 0.0009, effect size=0.55, BF=5.6) and Western savory (AUC = 0.5413, se = 0.037, *p* = 0.019, effect size = 0.32, BF = 1.28) food items. These MVPA findings further suggest that multisensory exposure has a stronger effect on Indian foods than on Western foods on Indian participants.

**Figure 6:**
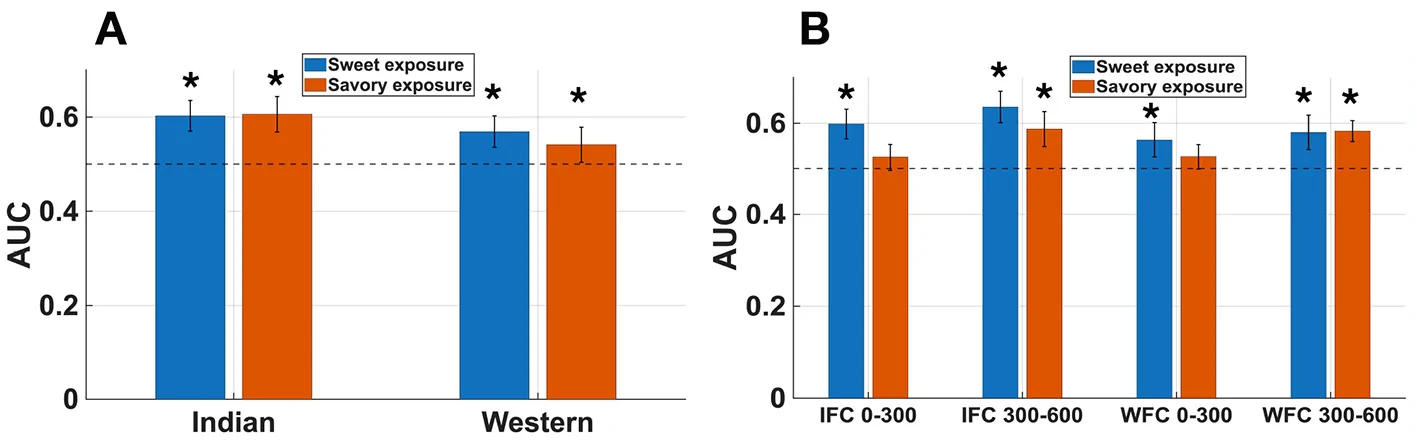
Classification AUC between pre-exposure and post-exposure after the channel selection method for both culture and both food types. **(A)** Full interval classification; **(B)** classification in two intervals. The AUC for Indian food exposures is higher than Western food exposures. * indicates that the AUC is significantly greater than chance.

Two window classification result is consistent with full-interval classification and univariate analyses. Indian sweet and savory give better classification results than Western sweet and savory. In the early time window (0–300 ms), AUC for sweet is significantly greater than chance for both Indian (AUC = 0.5985, se = 0.03, *p* = 0.00002, effect size = 0.82, BF = 23.51) and Western (AUC = 0.5634, se = 0.037, *p* = 0.007, effect size = 0.44, BF = 2.75), whereas the savory AUC is not significant for both Indian (AUC = 0.5249, se = 0.029, *p* = 0.09, effect size = 0.23, BF = 0.83) and Western (AUC = 0.5261, se = 0.02, *p* = 0.12, effect size = 0.27, BF = 0.81). Savory AUC became significant only in the later time window (300–600 ms) for Indian (AUC = 0.5874, se = 0.037, *p* = 0.0001, effect size = 0.61, BF = 5.22) and Western (AUC = 0.5830, se = 0.02, *p* = 0.0002, effect size = 1.04, BF = 47.27). AUCs for sweet are significant in the later window also for Indian (AUC = 0.6355, se = 0.03, *p* = 0.00001, effect size = 1.05, BF = 102.79) and Western (AUC = 0.5802, se = 0.037, *p* = 0.0007, effect size = 0.57, BF = 5.42).

### 3.5 Correlation analysis

Partial correlations between desire ratings and ERP components were computed and are presented in Table 4. With the exception of Western savory items, correlations were stronger post-exposure than pre-exposure. However, for savory foods from both cultures, the correlation between desire ratings and LPP amplitude decreased after exposure.

**Table 4:** Partial correlation analysis of ERP amplitudes with the desire and bid values at pre- and post-exposure conditions for Indian and Western food craving and for sweet and savory foods. High correlations (|*r*| ≥ 0.3) are given in bold.

Pre-exposure							Post-exposure				
Food type	Variables	Desire	bid	P200	N200	LPP	Desire	bid	P200	N200	LPP
IFC sweet	Desire	1	**0.56**	–0.25	–0.15	0.11	1	**0.61**	**–0.57**	**–0.52**	**0.39**
	Bid	**0.56**	1	0.05	0.01	–0.1	**0.61**	1	0.22	0.22	**–0.31**
	P200	–0.25	0.05	1	**–0.84**	0.23	**–0.57**	0.22	1	**–0.87**	0.27
	N200	–0.15	0.01	**–0.84**	1	**0.49**	**–0.52**	0.22	**–0.87**	1	**0.49**
	LPP	0.11	–0.1	0.22	**0.49**	1	**0.39**	**–0.31**	0.27	**0.49**	1
IFC savory	Desire	1	**0.52**	–0.098	–0.08	0.09	1	**0.52**	0.16	0.16	–0.06
	Bid	**0.52**	1	0.18	0.24	0.26	**0.52**	1	0.02	0.05	0.17
	P200	–0.098	0.18	1	**–0.86**	–0.02	0.16	0.02	1	**–0.88**	–0.19
	N200	–0.08	0.24	**–0.86**	1	0.17	0.16	0.05	**–0.88**	1	–0.14
	LPP	0.09	0.26	–0.02	0.17	1	–0.06	0.17	–0.19	–0.14	1
WFC sweet	Desire	1	**0.67**	–0.03	**–0.33**	–0.28	1	**0.68**	**0.30**	0.04	**–0.42**
	Bid	**0.67**	1	0.20	**0.5**	**0.4**	**0.68**	1	–0.02	0.25	**0.64**
	P200	–0.03	0.2	1	**–0.86**	–0.04	**0.30**	–0.02	1	**–0.83**	–0.10
	N200	**–0.33**	**0.5**	**–0.86**	1	–0.05	0.04	0.25	**–0.83**	1	–0.21
	LPP	–0.28	**0.41**	–0.04	–0.05	1	**–0.42**	**0.64**	–0.1	–0.21	1
WFC savory	Desire	1	**0.82**	–0.24	0.11	**–0.66**	1	**0.51**	–0.1	–0.13	–0.28
	Bid	**0.82**	1	0.009	**–0.43**	**0.85**	**0.51**	1	**–0.3**	**–0.32**	**0.51**
	P200	–0.24	0.009	1	**–0.85**	–0.1	–0.1	**–0.3**	1	**–0.96**	–0.04
	N200	–0.11	**–0.43**	**–0.85**	1	**0.34**	–0.13	**–0.32**	**–0.96**	1	0.001
	LPP	**–0.66**	**0.85**	–0.1	**0.34**	1	–0.27	**0.51**	–0.04	0.001	1

The bold *p*-values indicate that they are less than 0.05.

## 4 Discussion

We investigated the effect of both Western and Indian food in Indian population using food cues and multisensory exposure of sweet and savory food items. We observed that FCQ-S scores increased across both food types and cultures, indicating possible inducement of craving. In IFC, desire and bid increased significantly after both sweet and savory exposure but for WFC, desire and bid effect for sweet exposure were relatively weaker. Western savory exposure has no effect on desire and bid values on Indian participants. Through a linear mixed effect model we saw that desire and residual of reaction time and time (pre and post exposure) has significant main effect on bid prediction. Also there is an interaction effect between time and culture on bid prediction. The interaction plot shows that effect of multisensory exposure on bid values is more for Indian food than Western food. At the neural level, P200 and N200 amplitudes showed significant post-exposure increases for Indian sweet foods, whereas LPP amplitudes increased for savory foods from both Indian and Western categories. Given the modest sample size and the magnitude of these ERP effects, the univariate findings should nevertheless be interpreted cautiously. Importantly, the MVPA provided complementary evidence of exposure-related changes in distributed neural activity. Multivariate classification differentiated pre- and post-exposure conditions for all food categories, with generally higher AUCs for Indian than Western foods. The time-resolved MVPA further revealed a systematic temporal pattern: during the early 0–300 ms window, significant classification was observed for sweet but not savory foods, whereas during the later 300–600 ms window, significant classification was observed for both sweet and savory foods. This pattern partially converges with the ERP findings and suggests that exposure-related neural modulation possibly depend on both food category and processing stage. Finally, an exploratory partial-correlation analyses further suggested changes in the relationships between behavioral and ERP measures following exposure. Gender-related effects were generally limited, however, a food-type-by-gender interaction was observed for bid values in the savory-food condition.

Despite growing recognition of cultural influences on diet, much of the psychological and neuroscientific research on eating behavior has focussed on Western diet with high sugar high fat food items on Western population. This limitation has been widely discussed in behavioral science as the WEIRD problem, referring to the overrepresentation of participants from Western, Educated, Industrialized, Rich, and Democratic societies in psychological research (Henrich et al., 2010). Such sampling bias raises concerns about the generalizability of findings to other cultural contexts, particularly in domains such as eating behavior where cultural traditions play a central role (Counihan et al., 2013). Food varies widely across culture and people show increased preference for indigenous food compared to other cuisines (Einstein and Hornstein, 1970) however studies exploring cultural effect of food craving remains limited (Zellner et al., 1999; Osman and Sobal, 2006; Parker et al., 2003). Indigenous diets, shaped by local ecology, cultural identity, and long-standing traditions, often carry strong emotional significance and may evoke greater motivation than unfamiliar foods. Meanwhile, globalization has increased exposure to Western dietary patterns in non-Western societies—a shift known as the nutrition transition—characterized by a move toward processed foods high in sugar, fat, and refined carbohydrates (Popkin, 2017; Pingali, 2007). India is positioned uniquely for studying the interaction between traditional and Western food environments. Rapid urbanization and economic growth have led to significant changes in dietary patterns (Tak et al., 2019, 2022) and increase in consumption of ultra-processed foods (Tak et al., 2022; Law et al., 2019). For instance, a national mapping study of ultra-processed foods in India reported increasing availability and consumption of foods high in sugar, fat, and salt, raising concerns about their impact on diet-related health outcomes (Ghosh-Jerath et al., 2024). At the same time, qualitative studies of urban Indian consumers show that traditional dietary practices remain culturally meaningful and continue to influence food preferences and eating habits (Samaddar et al., 2020; Das and Priya, 2022).

We find that effect of Indian food is more prominent compared to Western food on Indian population as demonstrated by our behavorial and neural results. Food preferences in indigenous populations are strongly shaped by cultural familiarity and early exposure, leading to a persistent preference for traditional dietary patterns over Western alternatives (Rozin, 1996a, 2005; Kuhnlein and Receveur, 2007). In the Indian context, staple foods (e.g., rice-based, wheat-based, spiced preparations) are introduced early and consumed daily, leading to strong familiarity-driven preference over Western foods. Although India is undergoing a nutrition transition with increased availability of Western foods (Popkin, 2017), our results implies that Indian participants are more likely to prefer culturally familiar (indigenous) food. This aligns with findings from Kuhnlein and Receveur (2007) showing that culturally embedded foods carry higher emotional and symbolic value. Although a recent food cue study indicated no significant differences in self-report ratings of Chinese vs. Western food images on Chinese population, but it showed that Chinese food images eliparencited stronger activation in regions linked to cravings, taste perception, attention, reward, and visual processing (Xu et al., 2022). The complex sensory profiles and strong cultural associations of Indian foods further enhance their hedonic and motivational salience, suggesting that indigenous food choices may be preferred over Western foods despite increased exposure to the latter.

Exposure to sweet foods appears to be more pronounced than to savory foods across both Western and Indian cuisines, consistent with findings from the food craving literature. A substantial body of research indicates that sweet, sugar-rich foods exert a particularly strong influence on food craving by robustly activating dopaminergic reward pathways, reinforcing intake, and inducing addiction-like responses (Avena et al., 2008; Volkow et al., 2011). Sugar consumption elicits rapid dopamine release and engages both sensory and post-ingestive reward mechanisms, thereby strengthening learned preferences and craving (Thanarajah et al., 2019). Behavioral and neuroimaging studies consistently demonstrate that sweet foods are rated as more palatable and are more frequently craved than other taste categories and even surpass reward for addictive substance like cocaine (Lenoir et al., 2007), reflecting their heightened motivational significance (Hajnal and Norgren, 2004).

Gender differences in food craving are well-documented, with women consistently reporting higher craving frequency and intensity than men (Weingarten and Elston, 1991; Hallam et al., 2016). These differences extend to craving type, with women showing a stronger preference for sweet foods, particularly chocolate, whereas men more often prefer savory items (Zellner et al., 1999). Neuroimaging studies also indicate heightened activation in reward-related brain regions in women in response to food cues (Uher et al., 2006), suggesting that both biological and psychosocial factors contribute to gender differences in food craving. However, most of the studies are based on WEIRD population and cross-cultural research suggests that gender differences in craving are not universal, pointing to a significant role of sociocultural influences (Rozin, 2005). No significant gender effects were observed for desire, P200, N200, or LPP. However, a significant food-type-by-gender interaction was observed for bid values, with post-exposure increases in bids for savory foods being greater in males than in females. Thus, the present results do not support a general absence of gender-related modulation and instead suggest that gender effects, where present, may be specific to particular behavioral outcomes and food categories. Hence, the effect of gender on food craving—particularly for sweet foods—appears to be inconsistent and may not generalize across all populations.

MVPA provided evidence that multisensory exposure altered distributed neural response patterns even when corresponding changes were not always apparent in individual ERP amplitudes. Pre- and post-exposure conditions could be distinguished for all four food categories, with the highest AUCs observed for Indian sweet and savory foods and lower AUCs for Western sweet and savory foods. This pattern underscores the influence of cultural familiarity in shaping craving responses and aligns with both the behavioral and ERP findings. More importantly, the time-resolved analysis revealed a distinct temporal pattern. During the early time period, significant pre–post classification was observed for sweet foods from both Indian and Western categories, whereas classification for savory foods was not significantly above chance. During the later time window, significant classification was observed for both sweet and savory foods from both cultural categories. These findings suggest that exposure-related changes for sweet foods emerge relatively early, whereas detectable changes for savory foods become more apparent during later processing. The MVPA findings therefore complement, rather than simply duplicate, the univariate ERP results. The early sweet-food classification is broadly consistent with the P200/N200 effects observed for Indian sweet foods, while the emergence of significant savory-food classification in the later window is consistent with the observed LPP changes for savory foods. At the same time, significant MVPA effects in conditions without corresponding ERP amplitude differences indicate that multisensory exposure may alter distributed spatial-temporal neural patterns without necessarily producing a detectable change in the mean amplitude of a particular ERP component. Taken together, the ERP and MVPA findings point toward stimulus- and time-dependent modulation of neural food-cue processing following multisensory exposure. Early changes were most apparent for sweet foods, whereas later changes extended to both sweet and savory foods. The convergence across univariate and multivariate analyses is not complete, which is expected because ERP amplitudes characterize changes in selected components and electrode regions, whereas MVPA is sensitive to distributed patterns of neural activity. Accordingly, the two analyses should be viewed as complementary sources of evidence rather than as direct replications of one another.

P200 and N200 have been associated with early attentional allocation, perceptual discrimination, and stimulus evaluation, whereas the LPP is sensitive to sustained attention and the evaluative or motivational significance of salient stimuli (Carbine et al., 2018; Hajcak et al., 2010). However, these components are not uniquely associated with specific cognitive processes. Thus, the enhanced P200 and N200 responses to Indian sweet foods may reflect altered early attentional, perceptual, or evaluative processing, while the enhanced LPP for savory foods may be consistent with changes in sustained evaluative or motivational processing.

Several alternative explanations may contribute to these effects. Expectancy may have been altered through multisensory exposure, as sensory cues can generate expectations about a food's identity, sensory properties, and reward value (Piqueras-Fiszman and Spence, 2015). Visual food images can elicit differential ERP responses based on their semantic/nutritional properties, even when the relevant distinction is not simply low-level visual salience. (Toepel et al., 2009). Furthermore, the exposure procedure may have changed familiarity or novelty, which can influence neural responses to subsequently presented food cues through learning and previous experience (Kanoski and Boutelle, 2022). Finally, differences in visual attention, arising from both stimulus-driven visual salience and top-down factors such as prior experience and task demands, may contribute to the observed gaze distribution and consumer choice patterns (Motoki et al., 2021). Overall, our findings suggest a stimulus- and time-dependent modulation of food-cue processing following multisensory exposure. Rather than demonstrating a definitive dissociation between sensory salience and motivational evaluation, the combined ERP and MVPA results indicate that exposure may alter multiple stages of food-cue processing and that exposure-related neural patterns were detectable relatively early for sweet foods and more broadly across food types during later processing.

The present study examined cultural influences on food craving in an Indian sample by separately investigating sweet and savory items from Indian and Western cuisines using multisensory exposure. However, all stimuli consisted of high-calorie foods, which limits our ability to disentangle the specific role of caloric content in driving craving responses. The stimulus set was not objectively validated for several potentially relevant attributes, including perceived palatability, caloric value, macronutrient composition, emotional valence, and visual complexity.

The study did not assess several potentially relevant participant characteristics, such as socioeconomic status, trait food craving, and menstrual cycle status in female participants, which may have limited the comprehensiveness of participant characterization. In addition, the participants were drawn from a relatively homogeneous group consisting primarily of graduate students and research scholars of similar age. This relatively narrow sampling frame may limit the generalizability of the findings and cautions against interpreting the observed effects as broadly representative of the Indian population. Additionally, the relatively modest sample size limited the sensitivity of the study to small and moderate within-subject effects. As indicated by the sensitivity analysis, the study was primarily sensitive to relatively large effects; therefore, findings associated with smaller effect sizes, particularly some of the ERP effects, should be interpreted cautiously. The convergence of the ERP effects with the MVPA findings provides complementary evidence for exposure-related neural modulation, but does not eliminate the need for replication with a larger and more diverse cohort. The findings are based on an Indian sample and may not generalize to populations with different cultural food environments or dietary habits. The study focused on sweet and savory categories, and did not consider other taste dimensions (e.g., bitter, sour, umami), which may also differentially influence craving and neural responses. Future studies could additionally include a no-exposure control condition to permit a more rigorous evaluation of changes specifically attributable to multisensory exposure.

Further research could also extend this work by systematically manipulating both cultural context and caloric content—incorporating low- and high-calorie foods across sweet and savory categories within the same participants. Employing counterbalanced designs would allow for a more precise examination of how culture and energy density jointly influence craving in the Indian population.

## 5 Conclusion

The present study provides converging behavioral, electrophysiological, and multivariate evidence that food craving in an Indian population is associated with food type, cultural familiarity, and multisensory exposure. Behavioral responses following exposure differed across food categories, with stronger changes observed for Indian foods, suggesting that cultural familiarity may contribute to the modulation of motivational valuation following multisensory exposure. Western foods showed a more selective pattern, with significant increases observed primarily for sweet items.

The ERP findings further indicate that multisensory exposure alters the processing of food cues at different temporal stages. Increased P200 and N200 amplitudes were observed predominantly for Indian sweet foods, whereas enhanced LPP amplitudes were observed for savory foods across both Indian and Western categories. Complementary MVPA provided evidence of changes in neural response patterns following exposure, with enhanced pre- vs. post-exposure classification for sweet foods during both early and late windows and savory foods during the later window. Together, these findings indicate a pattern that is consistent with potentially different temporal sensitivities of neural responses to food type and cultural familiarity and that warrants further investigation in studies explicitly designed to test such temporal differences.

Overall, the findings demonstrate that food type and cultural context jointly contribute to variation in subjective and neural responses following multisensory food exposure. The results provide initial evidence for culturally dependent modulation of food-cue processing while highlighting the importance of examining food category and temporal dynamics when investigating craving and eating-related behavior. Future studies incorporating direct measures of expectancy, familiarity, visual attention, and motivational value, together with adequately powered designs and direct statistical comparisons of temporal effects, will be important for establishing the specific mechanisms underlying these exposure-related changes

## Data Availability

The raw data supporting the conclusions of this article will be made available by the authors, without undue reservation.
