# Supplementary material for: Food craving in a cultural context: behavioral and electrophysiological evidence from Indian and Western food sets
Source: Front Psychol. 2026 Oct 28;17:1940439. doi: 10.3389/fpsyg.2026.1940439 (PMC13751416; doi:10.3389/fpsyg.2026.1940439)
Supplement: Supplementary file 1 [file Supplementary_file_1.pdf]

Supplementary to Food Craving in a Cultural Context: Behavioral and Electrophysiological Evidence from Indian and Western Food Sets

Subhajit Das[a], Avishek Chatterjee[a,b], Satyaki Mazumder[a], and Koel Das*[a]

[a]Department of Mathematics and Statistics, IISER Kolkata, Mohanpur
[b]GlaxoSmithKline plc

July 2026

*koel.das@iiserkol.ac.in

1 Stimuli Set

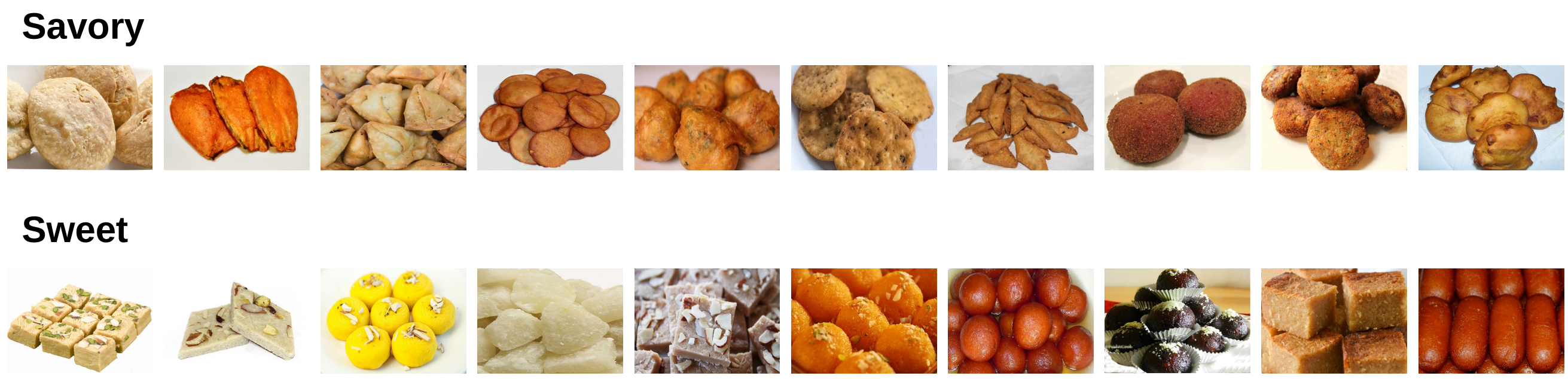

Figure S1: Stimuli set for the Indian food craving (IFC) experiment.

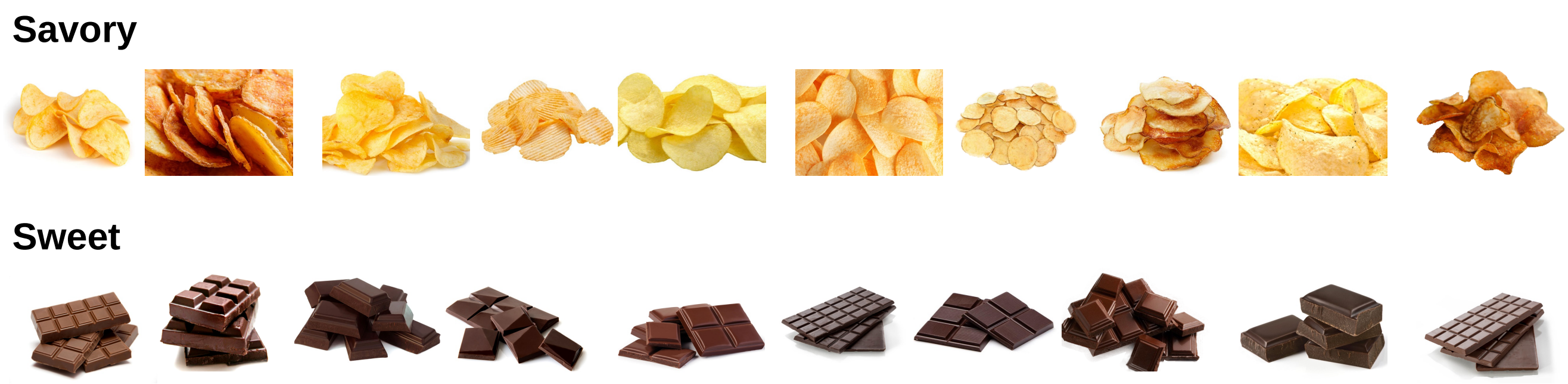

Figure S2: Stimuli set for the Western food craving (WFC) experiment

	F1	F2	F3	F4	F5	F6	F7	F8
F1	–	1.000	1.000	1.000	0.669	0.206	1.000	1.000
F2		–	1.000	1.000	1.000	0.464	1.000	1.000
F3			–	1.000	1.000	1.000	1.000	1.000
F4				–	1.000	0.460	1.000	1.000
F5					–	1.000	1.000	1.000
F6						–	1.000	1.000
F7							–	1.000
F8								–

Table S1: Holm-adjusted p-values for post-hoc test between the eight food categories' liking ratings taken during the screening of the Indian food craving experiment.

	F1	F2	F3	F4	F5	F6	F7	F8	F9	F10
F1	–	1.000	1.000	1.000	1.000	1.000	1.000	1.000	1.000	1.000
F2		–	1.000	1.000	1.000	1.000	1.000	1.000	1.000	1.000
F3			–	1.000	1.000	1.000	1.000	1.000	1.000	1.000
F4				–	1.000	1.000	1.000	1.000	1.000	1.000
F5					–	1.000	1.000	1.000	1.000	1.000
F6						–	1.000	1.000	1.000	1.000
F7							–	1.000	1.000	1.000
F8								–	1.000	1.000
F9									–	1.000
F10										–

Table S2: Holm-adjusted p-values for post-hoc test between the ten food categories' liking ratings taken during the screening of the Western food craving experiment.

2 LBRT Model with All Interactions

Here, we trained the bid reaction model by taking all the interaction effects to see the significance of the other interaction effects.

$$LBRT_{ij} = \alpha_0 + \alpha_1 D_{ij} + \alpha_2 T_{ij} + \alpha_3 F_j + \alpha_4 C_j + \alpha_5 (T_{ij} \times F_j) + \alpha_6 (T_{ij} \times C_j) + \alpha_7 (F_j \times C_j) + \alpha_8 (T_{ij} \times F_j \times C_j) + a_{0j} + \eta_{ij},$$

Coefficients of	Estimate	DF	t-value	p-value
intercept (α_0)	7.754	6227	101.11	< 0.001
desire (α_1)	0.054	6227	7.36	< 0.001
food type (α_2)	0.034	50	0.31	0.756
time (α_3)	-0.143	6227	-5.79	< 0.001
culture (α_4)	0.046	50	0.42	0.676
IbTF (α_5)	0.016	6227	0.46	0.647
IbFC (α_6)	0.087	50	0.55	0.583
IbTC (α_7)	-0.045	6227	-1.30	0.194
IbTFC (α_8)	0.029	6227	0.57	0.569

Table S3: LBRT full model fit results

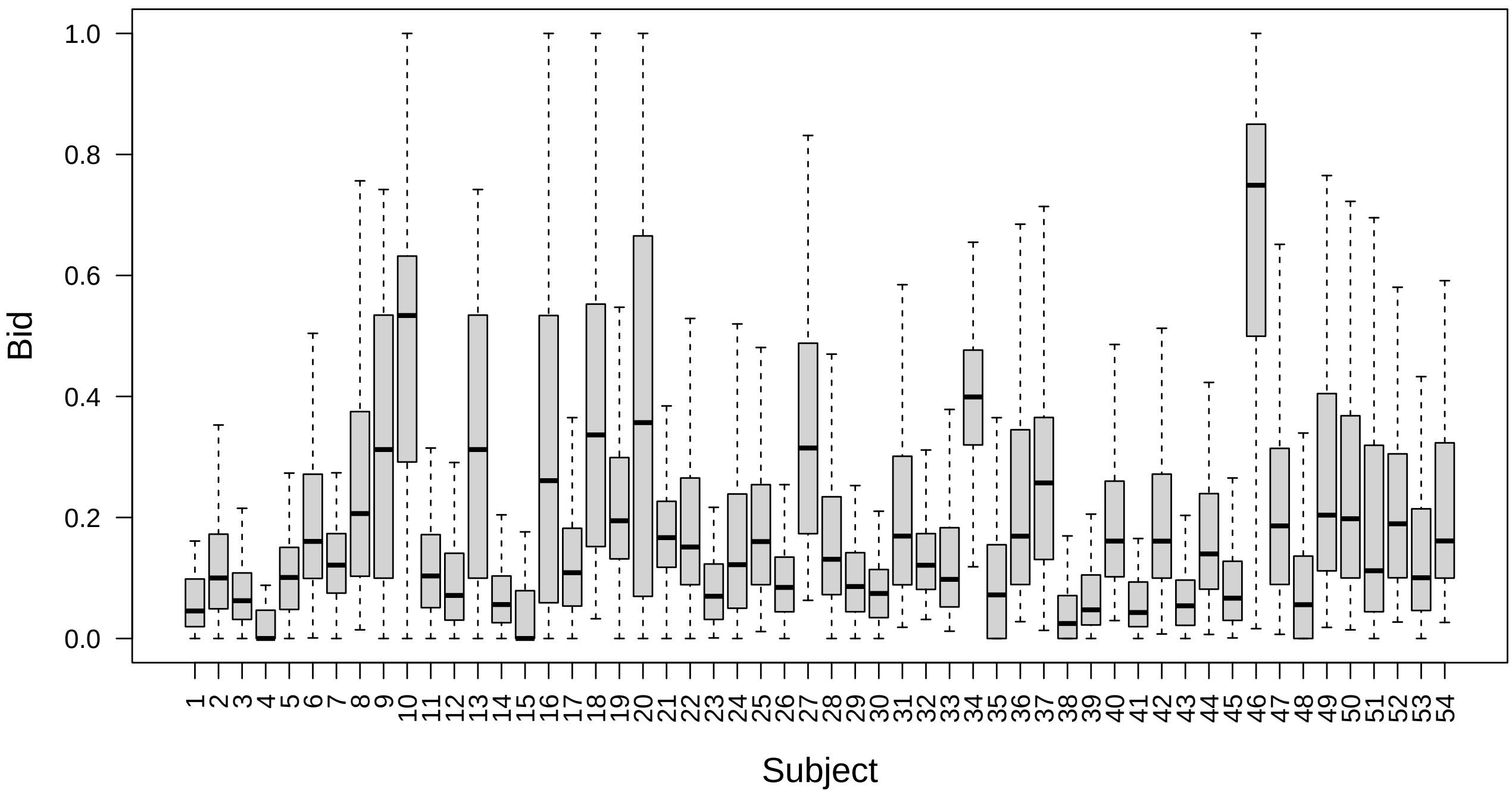

Figure S3: Boxplot of bid for all the subjects showing the variability across subjects.

3 Model Diagnosis

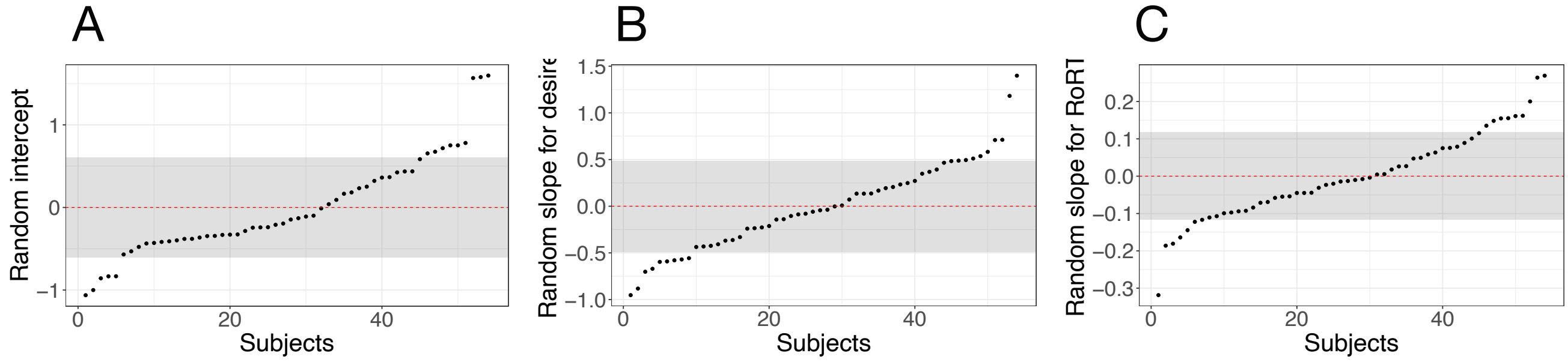

Figure S4: Caterpillar plot of (A) random intercepts; (B) random slope of desire; (C) random slope of Residual of Reaction Time (RoRT) of the model to show the subject-level variability in the model. The shaded region is the standard deviation of those estimated parameter values.

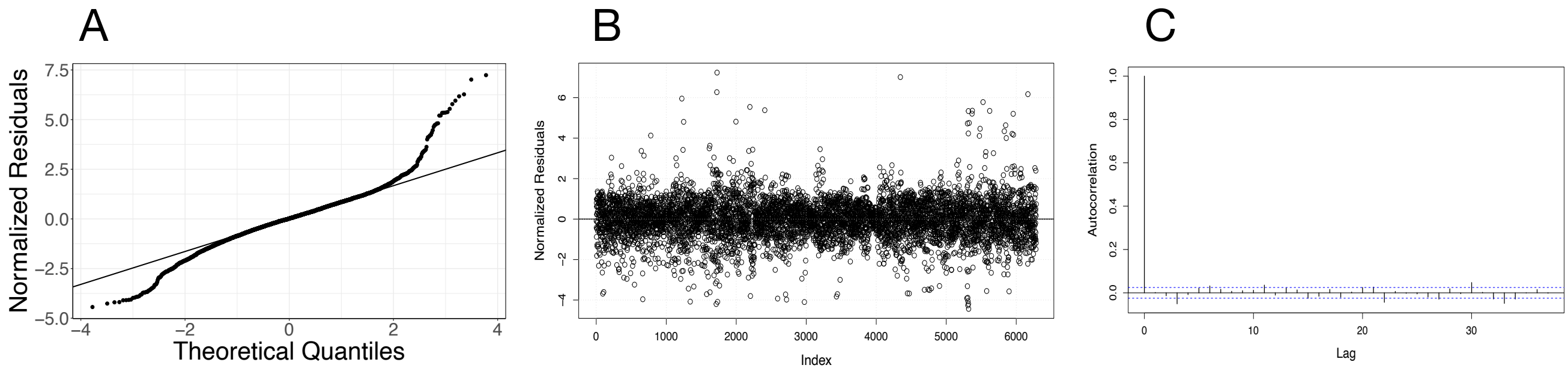

Figure S5: (A) Q-Q plot of the residuals of the model. (B) Residual plot of the model. (C) Autocorrelation plot of the residuals after fitting the model. The Q–Q plot indicates some departure from normality, particularly in the upper tail, suggesting mild positive skewness. The studentized residual plot shows no clear evidence of heteroscedasticity, and the ACF plot indicates no substantial residual autocorrelation.

4 Modeling to see if hunger rating immediately before experiment and BMI has any effect on the change of bid from pre- to post-exposure

**Coefficients of**	**Estimate**	**t-value**	**DF**	**p-value**
Intercept	0.0158	0.26	46	0.7947
Hunger	0.0079	0.51	46	0.6130
Culture	−0.0308	−0.37	46	0.7108
Food type	−0.0026	−0.03	46	0.9727
Hunger:Culture	−0.0050	−0.21	46	0.8379
Hunger:Food type	−0.0022	−0.11	46	0.9163
Culture:Food type	0.048	0.47	46	0.64
Hunger:Culture:Food type	-0.007	-0.23	46	0.812

Table S4: Results of linear mixed-effects model fit on change in bid values with hunger rating as a continuous variable and food type and culture as categorical variables.

**Variable**	**F-value**	**DoF**	**p-value**
Intercept	4.77	(1,46)	**0.0341**
Hunger	0.68	(1,46)	0.4151
Culture	2.06	(1,46)	0.1585
Food type	0.06	(1,46)	0.8046
Hunger Culture	0.56	(1,46)	0.4574
Hunger Food type	0.31	(1,46)	0.5801
Culture Food type	0.36	(1,46)	0.5498
Hunger Culture Food type	0.06	(1,46)	0.8120

Table S5: ANOVA results of the mixed-effect model fitted on change in bid values with hunger as a continuous variable and food type and culture as categorical variables.

**Coefficients of**	**Estimate**	**t-value**	**DF**	**p-value**
Intercept	−0.1366	−0.67	46	0.5077
BMI	0.0081	0.89	46	0.3781
Food type	0.3252	1.30	46	0.1990
Culture	−0.2475	−0.79	46	0.4313
BMI Food type	−0.0153	−1.37	46	0.1778
BMI Culture	0.0085	0.62	46	0.5412
Food type Culture	0.1461	0.38	46	0.7053
BMI Food type Culture	−0.0045	−0.26	46	0.7928

Table S6: Results of linear mixed-effects model fit on change in bid values with BMI as a continuous variable and food type and culture as categorical variables.

**Variable**	**F-value**	**DoF**	**p-value**
Intercept	5.19	(1,46)	**0.0273**
BMI	0.00	(1,46)	0.9654
Food type	0.02	(1,46)	0.9015
Culture	2.90	(1,46)	0.0953
BMI Food type	3.88	(1,46)	0.0548
BMI Culture	0.35	(1,46)	0.5598
Food type Culture	1.32	(1,46)	0.2562
BMI Food type Culture	0.07	(1,46)	0.7928

Table S7: ANOVA results of the mixed-effects model fitted on change in bid values with BMI as a continuous variable and food type and culture as categorical variables.

5 Gender Effect on All Measures

Effects	F-value	DoF	p-value
Culture	0.39	(1,46)	0.535
Food Type	0.085	(1,46)	0.772
Gender	0.825	(1,46)	0.368
Culture × Food Type	1.866	(1,46)	0.179
Culture × Gender	0.037	(1,46)	0.848
Food Type × Gender	0.487	(1,46)	0.489
Culture × Food Type × Gender	0.937	(1,46)	0.338

Table S8: 3-way factor ANOVA on the difference of desire values between post and pre-exposure conditions. From the table, it is visible that gender does not affect desire value.

Effects	F-value	DoF	p-value
Culture	3.223	(1,46)	0.0792
Food Type	0.042	(1,46)	0.8388
Gender	2.894	(1,46)	0.0957
Culture × Food Type	0.962	(1,46)	0.3318
Culture × Gender	1.349	(1,46)	0.2514
Food Type × Gender	6.738	(1,46)	**0.0126**
Culture × Food Type × Gender	0.106	(1,46)	0.7458

Table S9: 3-way factor ANOVA on the difference of bid values between post and pre-exposure conditions. Food type and gender has significant interaction. Post-hoc test shows that males' increment of bid after post-exposure is significantly better than females ($t = -3.107$, df=46, $p = 0.0032$, BF=13.96) for savory food exposure only.

Effects	F-value	DoF	p-value
Culture	0.077	(1,46)	0.782
Food Type	2.135	(1,46)	0.151
Gender	0.419	(1,46)	0.52
Culture × Food Type	0.109	(1,46)	0.742
Culture × Gender	0.457	(1,46)	0.502
Food Type × Gender	0.483	(1,46)	0.491
Culture × Food Type × Gender	0.089	(1,46)	0.766

Table S10: 3-way factor ANOVA on the difference of P200 ERP components between post and pre-exposure conditions. From the table, it is visible that gender does not affect P200 component.

Effects	F-value	DoF	p-value
Culture	0.866	(1,46)	0.357
Food Type	5.228	(1,46)	0.0269
Gender	1.037	(1,46)	0.3138
Culture × Food Type	0.643	(1,46)	0.4268
Culture × Gender	2.495	(1,46)	0.1211
Food Type × Gender	0.237	(1,46)	0.6286
Culture × Food Type × Gender	0.484	(1,46)	0.4899

Table S11: 3-way factor ANOVA on the difference of N200 ERP components between post and pre-exposure conditions. From the table, it is visible that gender does not affect N200 component.

Effects	F-value	DoF	p-value
Culture	1.837	(1,46)	0.1819
Food Type	3.222	(1,46)	0.0792
Gender	0.683	(1,46)	0.4128
Culture × Food Type	0.361	(1,46)	0.5508
Culture × Gender	0.724	(1,46)	0.3993
Food Type × Gender	1.217	(1,46)	0.2758
Culture × Food Type × Gender	1.926	(1,46)	0.1719

Table S12: 3-way factor ANOVA on the difference of LPP ERP components between post and pre-exposure conditions. From the table, it is visible that gender does not affect LPP component.

6 Change in bid model to see the block effect

Coefficients of	Estimate	DF	t-value	p-value
Intercept (γ_0)	0.031	485	1.933	0.053
block (γ_1)	0.001	485	0.929	0.353
food type (γ_2)	0.002	51	0.128	0.898
culture (γ_3)	-0.034	51	-1.905	0.062

Table S13: Results of the change in bid after post-exposure model to see the block effect.

7 Autocorrelations of the residuals after fitting the bid model without AR(1)

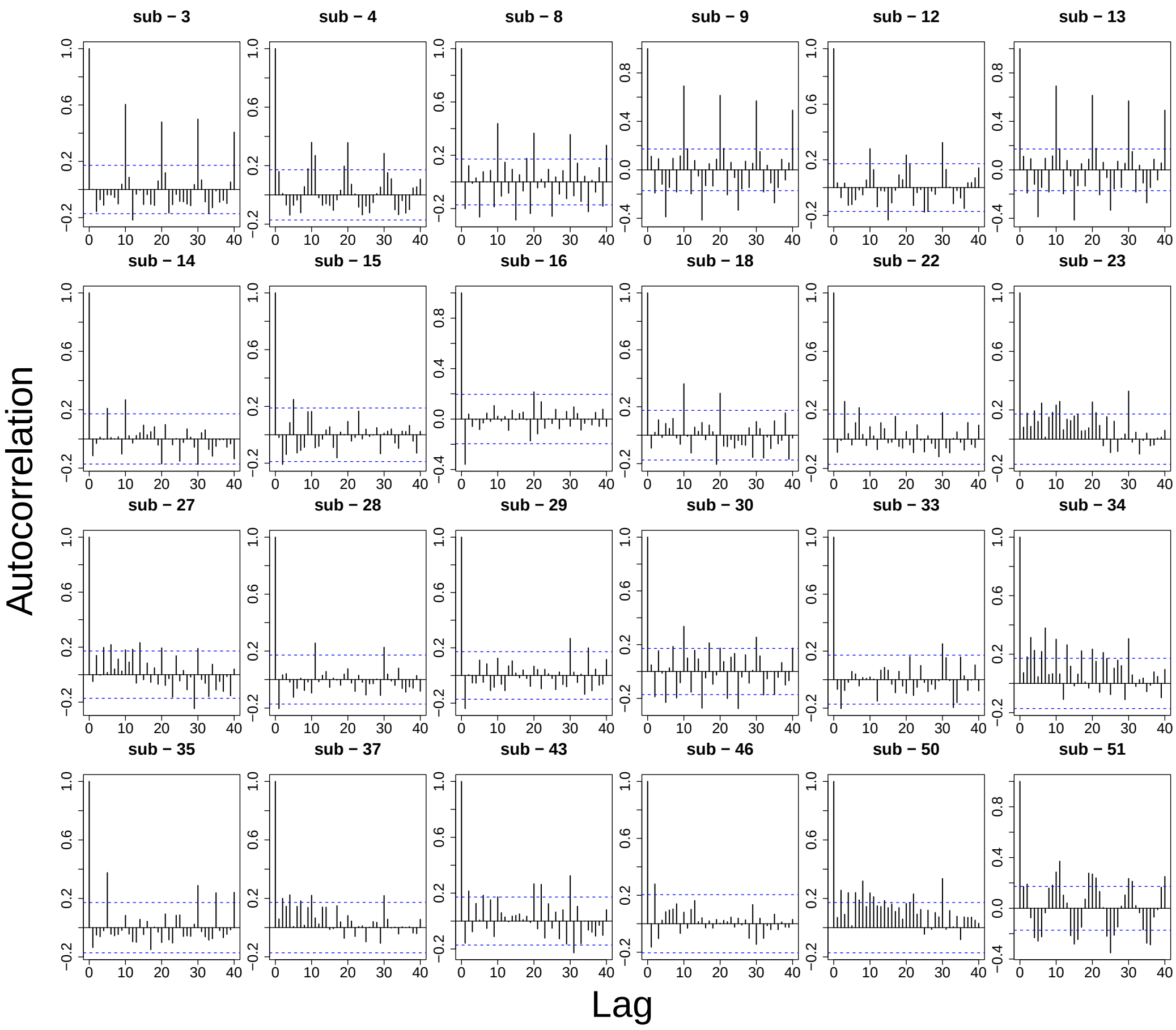

Figure S6: Autocorrelations of the residuals of some subjects after fitting the bid model without AR(1)

8 Topoplots

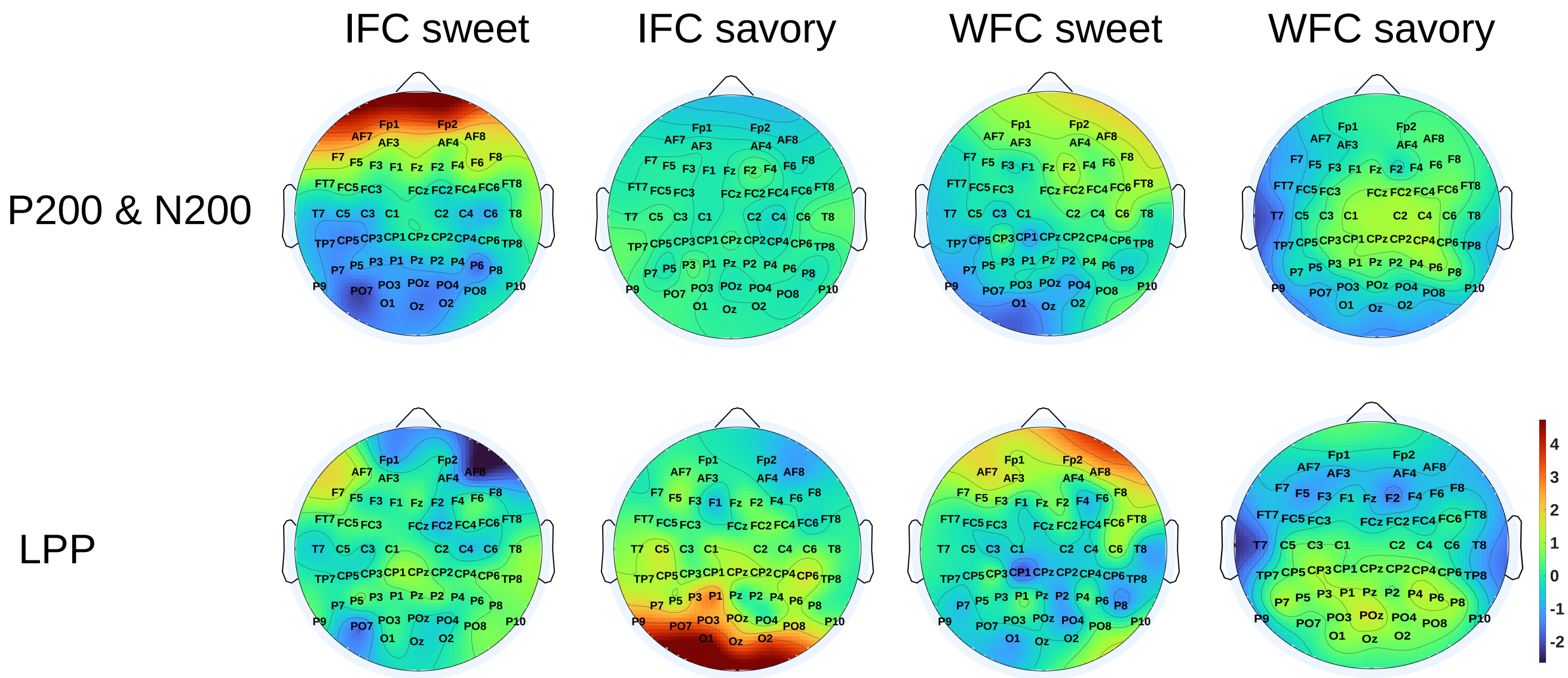

Figure S7: Topoplots of the difference waveform (post - pre-exposure) brain activity for P200, N200, and LPP in their corresponding time windows. For P200 and N200 in the 150-250 ms time window and for LPP in the 300-600 ms time window. Topoplots show the average brain activity in those intervals averaged across the corresponding subjects.

9 MVPA results

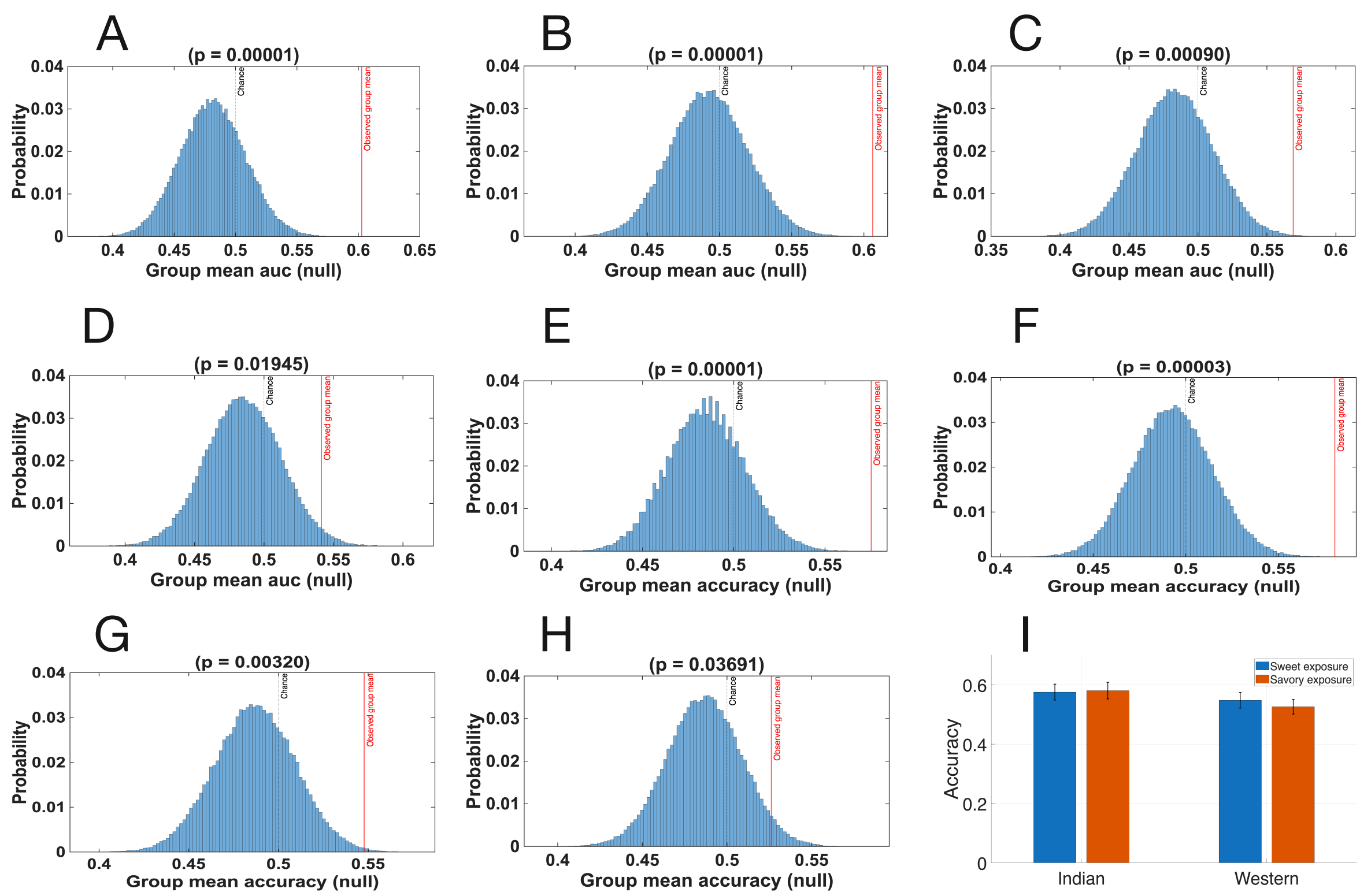

Figure S8: Permutation test results for (A) IFC sweet AUC, (B) IFC savory AUC, (C) WFC sweet AUC, (D) WFC savory AUC, (E) IFC sweet accuracy, (F) IFC savory accuracy, (G) WFC sweet accuracy, (H) WFC savory accuracy. (I) is the MVPA results for full interval for the accuracy measure.
